# Viral vector–mediated expression of Na_V_1.1, after seizure onset, reduces epilepsy in mice with Dravet syndrome

**DOI:** 10.1172/JCI159316

**Published:** 2023-06-15

**Authors:** Saja Fadila, Bertrand Beucher, Iria González Dopeso-Reyes, Anat Mavashov, Marina Brusel, Karen Anderson, Caroline Ismeurt, Ethan M. Goldberg, Ana Ricobaraza, Ruben Hernandez-Alcoceba, Eric J. Kremer, Moran Rubinstein

**Affiliations:** 1 Goldschleger Eye Research Institute, and; 2 Department of Human Molecular Genetics and Biochemistry, Faculty of Medicine, Tel Aviv University, Tel Aviv, Israel.; 3 Institut de Génétique Moléculaire de Montpellier, and; 4 PVM, BioCampus, CNRS, INSERM, University of Montpellier, Montpellier, France.; 5 Sagol School of Neuroscience, Tel Aviv University, Tel Aviv, Israel.; 6 Division of Neurology, The Children’s Hospital of Philadelphia, Philadelphia, Pennsylvania, USA.; 7 Department of Neurology and Neuroscience, The University of Pennsylvania School of Medicine, Philadelphia, Pennsylvania, USA.; 8 Gene Therapy and Regulation of Gene Expression Program, CIMA, University of Navarra. IdiSNA, Navarra Institute for Health Research, Pamplona, Spain.

**Keywords:** Neuroscience, Therapeutics, Epilepsy, Gene therapy, Neurodevelopment

## Abstract

Dravet syndrome (DS), an intractable childhood epileptic encephalopathy with a high fatality rate, is typically caused by loss-of-function mutations in one allele of *SCN1A*, which encodes Na_V_1.1, a 250-kDa voltage-gated sodium channel. In contrast to other epilepsies, pharmaceutical treatment for DS is limited. Here, we demonstrate that viral vector–mediated delivery of a codon-modified *SCN1A* open reading frame into the brain improves DS comorbidities in juvenile and adolescent DS mice (*Scn1a*^A1783V/WT^). Notably, bilateral vector injections into the hippocampus and/or the thalamus of DS mice increased survival, reduced the occurrence of epileptic spikes, provided protection from thermally induced seizures, corrected background electrocorticographic activity and behavioral deficits, and restored hippocampal inhibition. Together, our results provide a proof of concept for the potential of *SCN1A* delivery as a therapeutic approach for infants and adolescents with DS-associated comorbidities.

## Graphical abstract

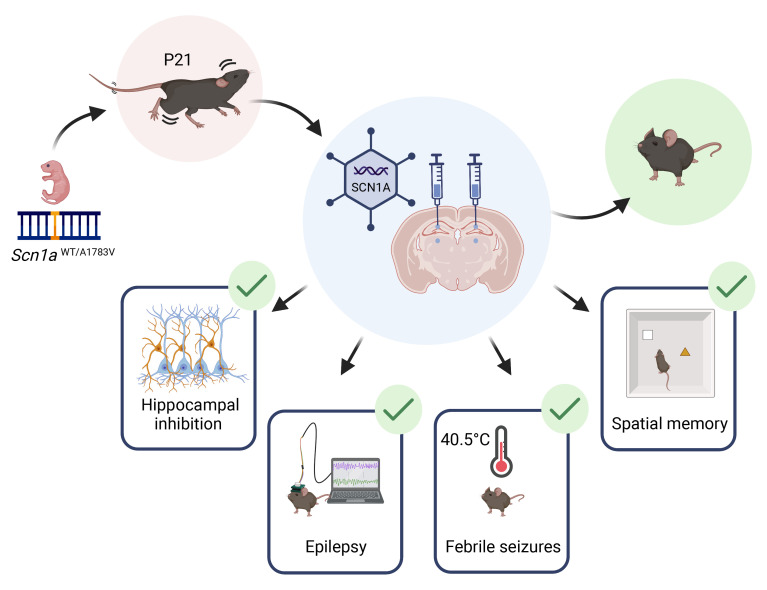

## Introduction

Dravet syndrome (DS) is a rare and severe form of developmental epileptic encephalopathy. Infants with DS appear to develop normally during the first 6 months of life, and then subsequently start to exhibit febrile seizures. During the following months, recurrent refractory spontaneous seizures become increasingly more frequent and global developmental delays begin. During the early school years, which represent the chronic phase of the disease, the frequency and duration of seizures decline, but the nonepileptic comorbidities persist (1–3).

The vast majority of DS cases are caused by de novo mutations in one allele of *SCN1A*. These mutations cause reduced or complete loss of function and therefore insufficient activity of the voltage-gated sodium channel, Na_V_1.1 (4). Thus, a strategy in which Na_V_1.1 activity is restored in central nervous system (CNS) neurons can represent a useful therapeutic approach, regardless of the underlying *SCN1A* mutation.

One option for therapy is to provide exogenous Na_V_1.1 via delivery of the *SCN1A* open reading frame (ORF). However, due to the size of the *SCN1A* ORF (~6 kbp), commonly used vectors, such as adeno-associated virus (AAV), are poorly adapted. In an attempt to circumvent the size obstacle, strategies for DS therapy have included (i) enhanced expression of the endogenous *Scn1a* via transcriptional activation (5–8); (ii) overexpression of *SCN1B*, which encodes Na_V_β1, an auxiliary subunit that increases Na_V_1.1 channel complex efficacy (9); and, (iii) antisense oligonucleotide–mediated downregulation of *SCN8A* (10). While these approaches showed proof of principle in mouse models of DS, the therapeutic potential was manifested only when administered soon after birth, during the asymptomatic, pre-epileptic stage (5, 7, 9, 10). As clinical diagnoses are rarely confirmed prior to progression to recurrent spontaneous seizures, therapies able to reduce the epilepsy and protect from sudden unexpected death in epilepsy (SUDEP), following the onset of severe intractable seizures, remain a critical unmet need. Recent studies indicate that conditional activation of *Scn1a* in adult mice can reverse DS symptoms (11). In this study, activation was mediated by Cre recombinase–induced removal of a floxed stop signal that was inserted into *Scn1a*, and therefore is not translatable into clinical use. Nonetheless, these results provide validation that restoration of Na_V_1.1 activity, in critical brain regions, can reduce DS symptoms.

In contrast to AAV vectors, helper-dependent (HD) adenovirus vectors can harbor up to 37 kb of exogenous sequence and allow rapid transgene expression after injection (12–14). The HD vector cloning capacity allows the option of multiple cassettes with large transcription regulatory sequences. Moreover, canine adenovirus type 2 (CAV-2) vectors provide numerous advantages for gene transfer to the CNS (14–16). First, by using the coxsackievirus and adenovirus receptor (CAR) as an attachment molecule, the expression of which is found primarily in neurons in the brain parenchyma (17), CAV-2 vectors preferentially transduce neurons (18). Second, with its robust retrograde axonal transport, local injections can lead to expression across connected brain regions (18). Third, like AAV vectors, the injection of E1/E3-deleted or HD CAV-2 vectors generates efficient, long-term transgene expression in the CNS of rodents, dogs, and nonhuman primates (16, 19).

Here, we demonstrate that CAV-2–mediated delivery of a codon-modified *SCN1A* ORF into the brain of adolescent DS mice significantly reduces epileptic spike frequency and increases the temperature threshold for febrile seizures. In juvenile mice, this treatment improves the survival of the mice, reduces the occurrence of spontaneous seizures and epileptic spike frequency, restores hippocampal inhibition, increases the temperature threshold for febrile seizures, and corrects behavioral deficits. Together, our results demonstrate that neuronal delivery of an expression cassette encoding Na_V_1.1 is a promising therapeutic approach for DS.

## Results

### Transcriptionally targeted transgene expression.

The current dogma is that many DS-associated symptoms are caused by inhibitory neuron dysfunction (20). However, others have postulated that reduced Na_V_1.1 activity in excitatory neurons contributes to DS comorbidities (21–23). Moreover, the hippocampus plays a key role in DS pathophysiology, and local deletion of *Scn1a* in excitatory and inhibitory neurons in this region can cause seizures (24, 25). We therefore initiated assays to determine whether we could target transgene expression to different neuronal populations in the hippocampus. Because targeted infection of these neuronal subtypes is not yet technically feasible, we opted for transcriptional control of the expression cassette. To this end, we prepared CAV-2 vectors containing a fluorescent protein driven by (i) the nonspecific CAG promoter (cytomegalovirus enhancer, chicken β-actin promoter and rabbit β-globin splice acceptor site) that should drive expression in all vector-transduced cells; (ii) the human synapsin promoter (hSyn), which drives transgene expression in most neuronal populations; (iii) the neuron-specific enolase (*NSE*) promoter, which also drives transgene expression in excitatory and inhibitory neurons (26); and (iv) the *Dlx5*/*6* promoter (*Dlx5* and *Dlx6* encode 2 homeobox transcription factors expressed by developing and mature GABAergic interneurons), which, when incorporated into a viral vector, can lead to preferential expression in inhibitory neurons (27, 28) (see Supplemental Figure 1 for schematic of expression cassettes; supplemental material available online with this article; https://doi.org/10.1172/JCI159316DS1).

**Figure 1:**
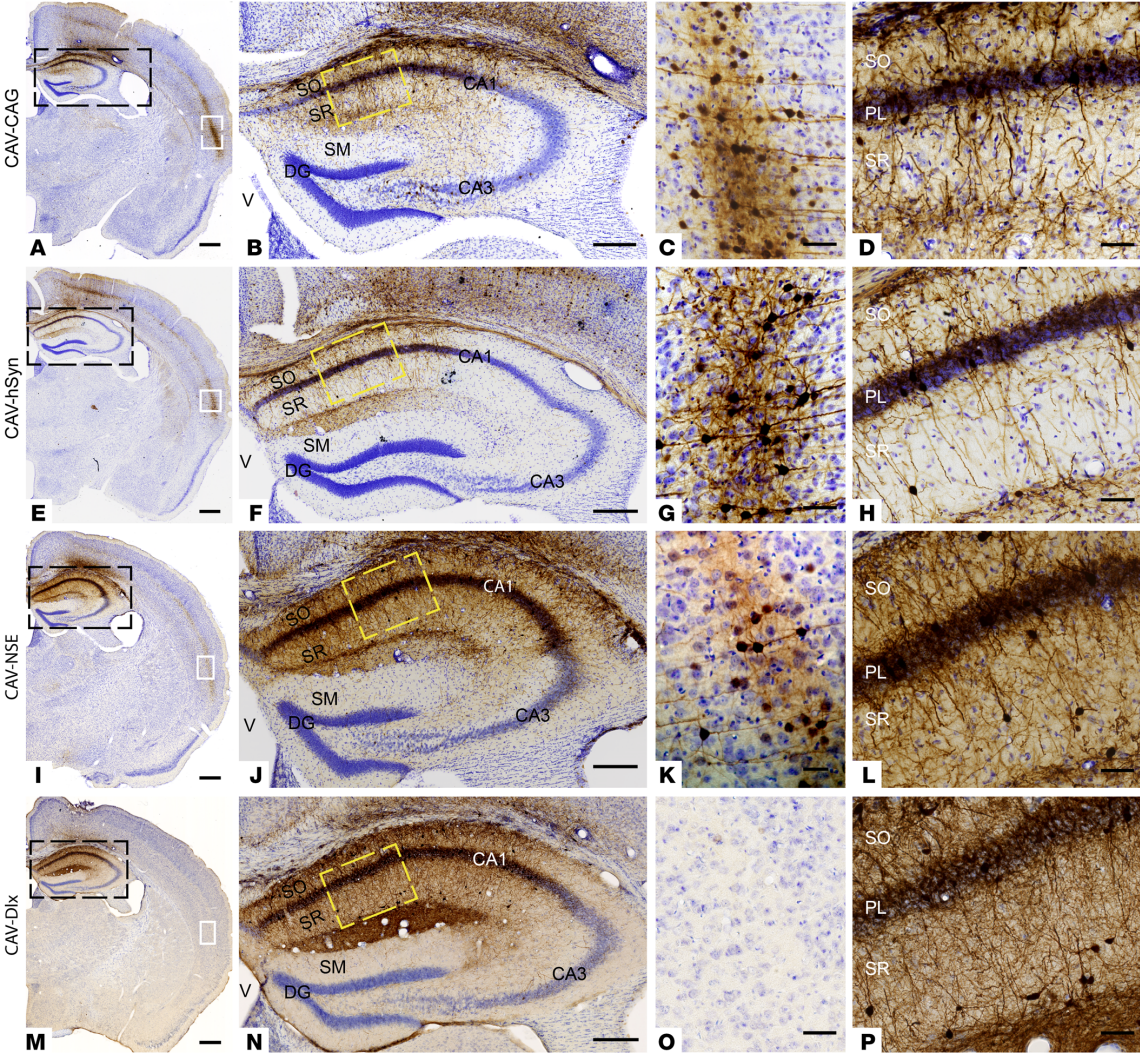
Transcriptional control of transgene expression in CAV-2 vectors following injection into the mouse hippocampus. CAV-2 vectors containing various promoters upstream of an mCitrine ORF were generated. Physical particles (1 × 10^9^) of each vector were injected bilaterally into the hippocampus of adult mice (*n* = 5 mice/vector). mCitrine expression is shown by immunohistological DAB staining (dark brown). All the sections were counterstained with cresyl violet. The left-hand column shows a representative hemisphere, the second column shows the hippocampus (magnification of black box in the first column), the third column shows transgene expression in the neocortex (magnification of white box in the first column), and the fourth column shows transgene expression in the CA1 region (magnification of yellow box in the second column). (**A**–**D**) *CAG* promoter–driven mCitrine expression. (**E**–**H**) hSyn-driven mCitrine expression. (**I**–**L**) *NSE*-driven mCitrine expression. (**M**–**P**) *Dlx*-driven (*Dlx5*/*6*-driven) mCitrine expression. PL, pyramidal cell layer; SO, stratum oriens; SR, stratum radiatum; V, ventricle; CA1, hippocampal CA1 region. Scale bars: 1 mm (**A**, **E**, **I**, and **M**) 250 μm (**B**, **F**, **J**, and **N**), 10 μm (**C**, **G**, **K**, and **O**), and 50 μm (**D**, **H**, **L**, and **P**).

The above-mentioned vectors were then injected into the CA1 region of the hippocampus of adult mice. The CAG promoter generated widespread transgene expression at the site of injection, where we observed mCitrine immunoreactive somata and fibers in different layers of the CA1 region (Figure 1, A, B, and D) and, due to the retrograde transport of CAV-2, in neuronal somata in multiple neocortical areas (Figure 1, A and C). Like the CAG promoter, the hSyn and *NSE* promoters led to somata expression in excitatory and inhibitory neurons (based on location and morphology) at the injection site (Figure 1, E–L). mCitrine^+^ somata and fibers were present in the different layers of the CA1 region, in the subiculum, and neocortical layers IV, V, and VI (with both promoters), layer II (when using the hSyn promoter), entorhinal cortex layers II and III, and fibers in the commissure, dorsal thalamus, and striatum (Supplemental Figure 2). Conversely, the *Dlx5*/*6* promoter produced localized transgene expression principally in fibers and soma in different layers of the CA1, the location and morphology of which were consistent with interneurons (Figure 1, M–P).

**Figure 2:**
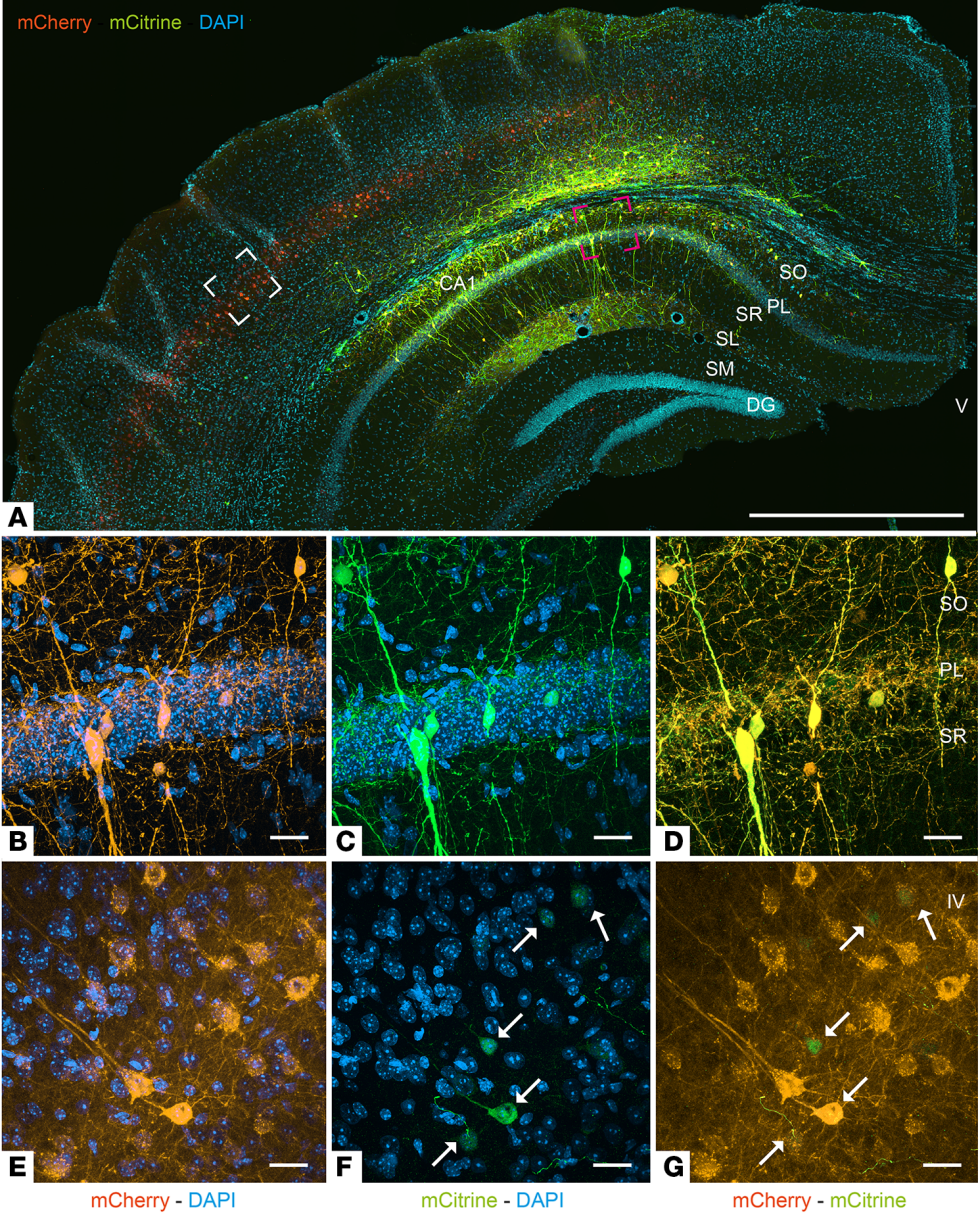
CAV-NSE expression is robust in the hippocampus and moderate in the neocortex. CAV-hSyn-mCherry and CAV-NSE-mCitrine vectors were coinjected bilaterally into the hippocampus of adult mice (*n* = 3 mice). (**A**) Micrograph showing the expression of mCitrine (green) and mCherry (orange) in the hippocampus and adjacent cortical regions. (**B**–**D**) High magnification of the red square in **A**, showing the colocalization of mCitrine and mCherry in somata and fibers in the layers of the CA1. (**E**–**G**) High magnification of white square in **A**, showing neocortical neurons expressing mCherry, mCitrine, or both (white arrows). PL, pyramidal cell layer; SO, stratum oriens; SR, stratum radiatum; IV, neocortex layer IV; V, ventricle. Scale bars: 1 mm (**A**) and 10 μm (**B**–**G**).

We reasoned that an effective and safe approach for DS therapy would require a neuron-specific promoter that drives Na_V_1.1 expression in both inhibitory and excitatory neurons in the hippocampus, and moderate expression in nonhippocampal glutamatergic neurons that project to the injection site. As the CAG promoter is not neuron specific (29), and the *Dlx5*/*6* promoter led to restricted hippocampal expression, these considerations focused our choice on the hSyn or the *NSE* promoters. To compare hSyn and *NSE* promoters, we generated CAV-hSyn-mCherry and coinjected it with CAV-NSE-mCitrine into the mouse hippocampus. While we found overlapping expression in the CA1 region (Figure 2, A–D), the hSyn promoter led to more neocortical neurons expressing the transgene than the *NSE* promoter (Figure 1, E and I, Figure 2, and Supplemental Figure 2). While transduction of more neurons/brain regions may be advantageous, the impact of the exogenous Na_V_1.1 activity in glutamatergic neocortical neurons is uncertain. Together, these data suggested that the *NSE* promoter, which leads to robust neuronal expression in the hippocampus and moderate, more restricted expression in the glutamatergic neocortical hippocampal projecting neurons, was a pragmatic choice.

**Figure 3:**
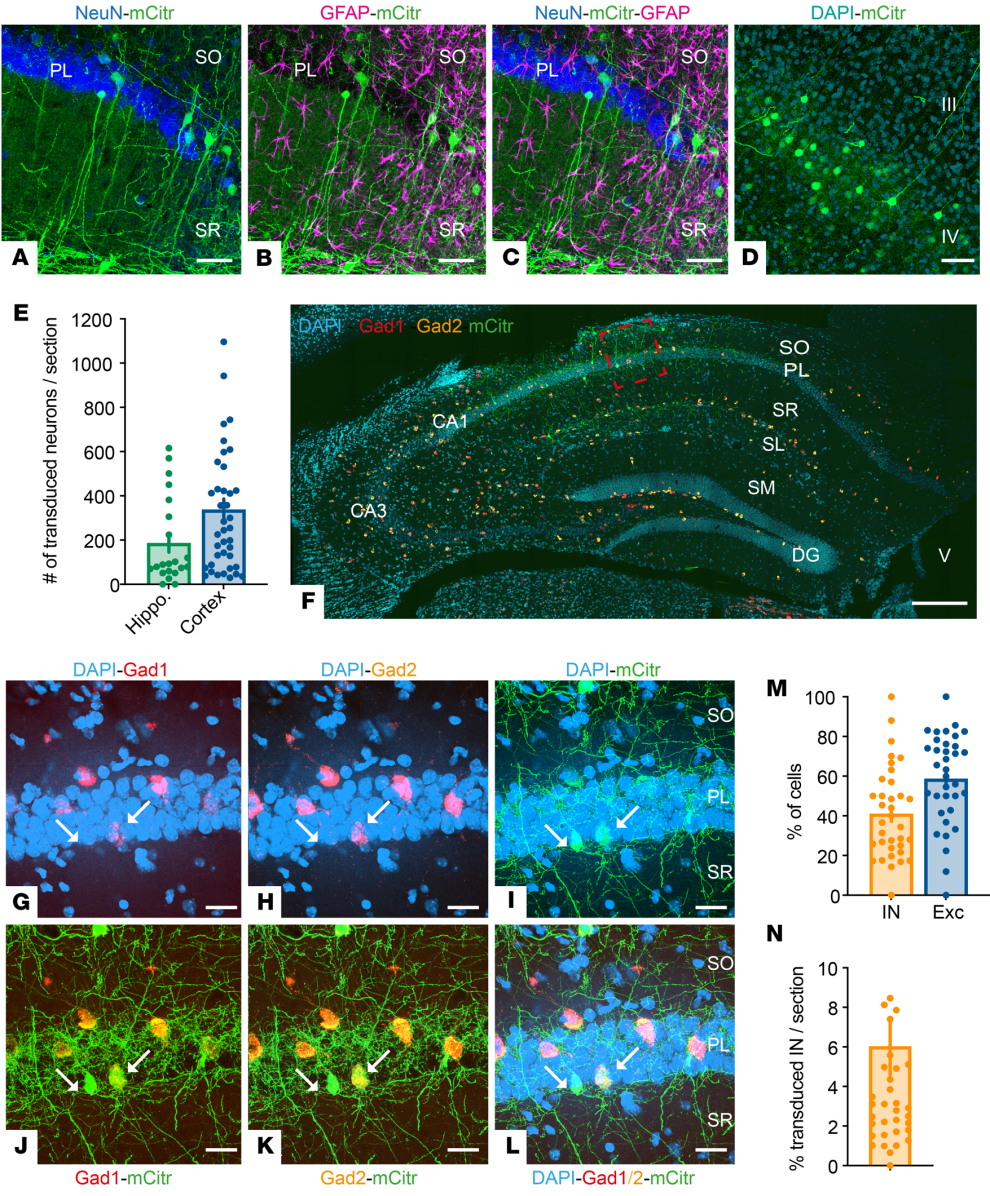
CAV-NSE transduces excitatory and inhibitory neurons. (**A**–**C**) Photomicrographs showing the presence of mCitrine (green) in neurons labeled with NeuN (blue), but not in glial cells labeled with GFAP (magenta) in different layers of the CA1 region. (**D**) High magnification of a neocortical region showing the presence of mCitrine in layer IV neurons. (**E**) The number of transduced neurons in the hippocampus and the cortex per 35-μm-thick section. *n* = 5 mice, 7–9 sections/mouse. (**F**–**L**) Representative sections of a hippocampus labeled with RNAScope (*Gad1* in red, *Gad2* in orange), mCitrine (green), and DAPI (cyan). (**G**–**L**) High magnifications of the red square in **F**, showing cells colocalizing mCitrine and *Gad1*/*2* in different layers of the CA1 region (white arrows). (**M**) The percentage of inhibitory and excitatory neurons out of the transduced, mCitrine^+^ cells. (**N**) The percentage of transduced inhibitory neurons (IN) out of the residing inhibitory neurons (*n* = 5 mice, 3–10 sections/mouse). PL, pyramidal cell layer; SO, stratum oriens; SR, stratum radiatum; SM stratum moleculare; SL, stratum lacunose; DG, dentate gyrus. Scale bars: 50 μm (**A**–**D**), 1 mm (**F**), and 10 μm (**G**–**L**).

Quantification of the number of mCitrine^+^ neurons following injection of CAV-NSE-mCitrine demonstrated an average of approximately 180 hippocampal neurons/35-μm-thick section (>10,000 neurons throughout the structure) and approximately 340 cortical neurons/35-μm-thick section (>20,000 neurons in the neocortex) (Figure 3, A–E). Of note, these data do not include transduced neurons in noncortical regions that project to the hippocampus. Importantly, transgene expression was robust for at least 9 months after injection (Supplemental Figure 3).

Finally, using RNAscope for *Gad1* and *Gad2* transcripts, we quantified the percentage of transduced GABAergic cells in the hippocampus. We found that over 40% of the transduced cells were *Gad1*/*2*^+^ (i.e., inhibitory neurons; Figure 3, F–N), with a mean efficacy of approximately 6% of the inhibitory neurons in each hippocampal section (Figure 3N). As inhibitory neurons represent approximately 15% of the hippocampal neurons (30), we concluded that an *NSE* promoter–driven cassette in a CAV-2 vector leads to preferential expression in inhibitory neurons.

### CAV-SCN1A–mediated Na_V_1.1 activity.

**Figure 4:**
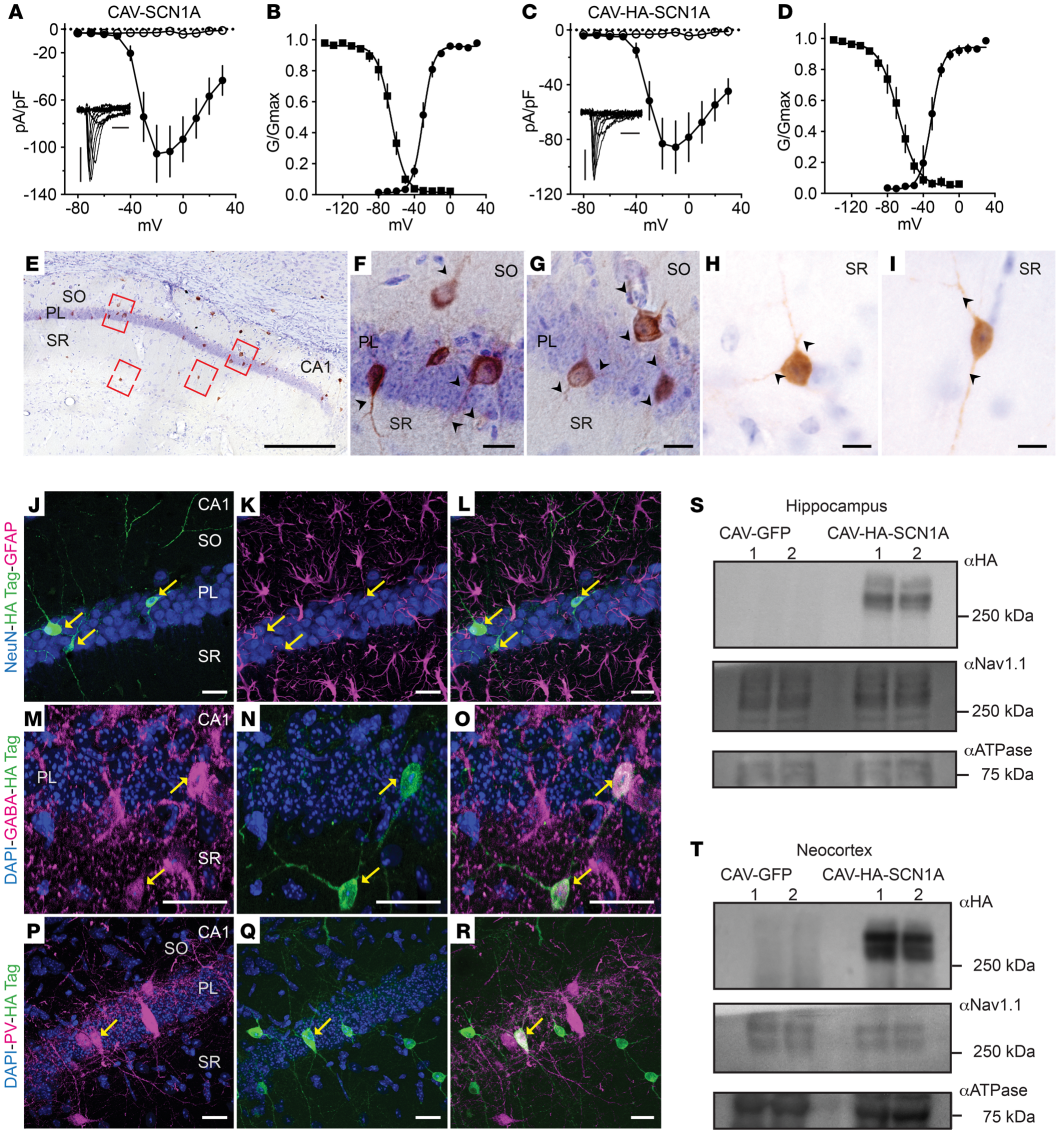
CAV-2–mediated Na_V_1.1 activity and in vivo location. (**A** and **B**) Voltage-current relationship (**A**) and the biophysical properties (**B**) of sodium currents following expression of CAV-SCN1A in DK cells. The half voltage of activation/inactivation was –30.4 ± 0.7 mV, and –65.73 ± 0.9 mV, respectively. (**C** and **D**) Sodium currents following expression of CAV-HA-SCN1A in DK cells. The half voltage of activation/inactivation was –30.6 ± 1.2 mV and –67.8 ± 1.8 mV, respectively. Insets in **A** and **C** show representative sodium currents (calibrators: 500 pA, 2 ms). The empty symbols depict the currents from DK cells infected with CAV-GFP (*n* = 10, presented in **A** and **D**), and closed symbols depict CAV-SCN1A (*n* = 9) or CAV-HA-SCN1A (*n* = 7). (**E**–**I**) CAV-HA-SCN1A was injected bilaterally into the hippocampus of adult mice. HA immunoreactivity is shown in brown; all sections were counterstained using cresyl violet. (**E**) Low-magnification micrographs showing the presence of HA-immunoreactive cells in different layers of the CA1 region, 2 weeks after injection. (**F**–**I**) High magnification of the red boxes in **E** showing HA-immunoreactive cells in the layers of the CA1 region. The immunoreactivity is present in the somata and in the fibers (arrow heads). (**J**–**L**) Confocal images showing mCitrine (green) in NeuN^+^ cells, but not in GFAP^+^ cells (magenta) in the CA1 of an adult mouse. (**M**–**O**) High magnification of the CA1 showing the presence of mCitrine (green) in GABAergic cells (magenta, yellow arrows). (**P**–**R**) *Z*-stack of confocal images showing mCitrine^+^ (green) in parvalbumin (PV) immunoreactive cells (magenta, yellow arrows). Scale bars: 1 mm (**E**), 10 μm (**F**–**I**), 25 μm (**J**–**L**), 50 μm (**M**–**O**), and 25 μm (**P**–**R**). PL, pyramidal cell layer; SO, stratum oriens; SR, stratum radiatum. (**S** and **T**) Ten days after hippocampal injection of CAV-GFP (*n* = 2 mice) or CAV-HA-SCN1A (*n* = 2 mice), the hippocampi (**S**) and neocortex (**T**) were isolated and membrane bound proteins were extracted. Western blot analyses using anti-HA and anti-Na_V_1.1 are shown. ATPase was used as an internal control.

In addition to the size of the *SCN1A* ORF, the native sequence is prone to rearrangement when subcloned into a plasmid and propagated in *E*. *coli* (20). However, codon modification can enhance the stability and thereby facilitate the creation of vectors (29). To be able to readily amplify plasmids containing an *SCN1A* ORF, we generated a codon-optimized sequence and then manually screened for, and eliminated, 10- to 15-bp repeat sequences found primarily in the regions encoding the 24 transmembrane domains of Na_V_1.1. CAV-2 vectors containing the *NSE* promoter driving expression of the codon-modified *SCN1A* ORF (CAV-SCN1A) and a codon-modified *SCN1A* ORF with a C-terminal hemagglutinin (HA) tag on Na_V_1.1 (CAV-HA-SCN1A), were generated and purified as previously described (31) (Supplemental Figure 1). The 9–amino acid HA tag allowed us to identify exogenous Na_V_1.1 by anti-HA immunohistochemistry. To control for the generation of functional Na_V_1.1 channels, we incubated CAV-SCN1A or CAV-HA-SCN1A with DK cells (31). Voltage-gated sodium currents with the biophysical properties that are characteristic of Na_V_1.1 were indistinguishable between native and HA-tagged proteins (Figure 4, A–D) (32). Although we found no functional difference between the native and HA-tagged Na_V_1.1, the vector encoding native Na_V_1.1 was used to examine the therapeutic effect (see below).

Following the injection of CAV-HA-SCN1A into the hippocampus of adult mice, we found robust HA immunoreactivity in the somata and projections of multiple types of hippocampal neurons, including neurons that were also immunoreactive for GABA or parvalbumin (Figure 4, E–R). Moreover, Western blot analyses of dissected hippocampi and neocortex were consistent with robust HA-tagged Na_V_1.1 expression in multiple regions due to CAV-2 retrograde transport to efferent regions and transduction of glutamatergic hippocampal projecting neurons (Figure 4, S and T; see complete unedited blots in the supplemental material). Of note, the differences in the intensity of DAB staining and overall distribution of membrane-targeted HA-SCN1A (Figure 4, E–R), compared with that of cytoplasm-filling GFP (Figure 1, I–L, and Figures 2 and 3), likely underestimate HA-SCN1A levels, due to the difficulties in detection of Na_V_ channels in fixed tissue (33, 34), and the use of a monoclonal anti-HA versus polyclonal anti-GFP antibodies.

### CAV-SCN1A injections revert epileptic phenotypes in adolescent DS mice.

There are multiple mouse models of DS that faithfully reproduce the hallmarks of DS pathology (21, 35–42). Most DS mice display age-dependent progression of the severity of the epilepsy, with spontaneous seizures that begin around postnatal day 18 (P18), and premature death that peaks during the fourth week of life. Surviving DS mice enter a chronic stage, in which the mortality and frequency of spontaneous convulsive seizures are reduced (39). The genetic background dramatically affects the severity of the epileptic phenotype (22, 36, 43, 44). Here, we used a DS mouse harboring a disease-causing missense mutation (*Scn1a*^A1783V/WT^) on the pure C57BL/6J background. All DS *Scn1a*^A1783V/WT^ mice experience spontaneous seizures, with an overall mortality rate of over 50%, and thermally induced seizures that occur within the range of clinical febrile seizures (39, 42, 45–47). Moreover, *Scn1a*^A1783V^ causes Na_V_1.1 loss of function (48), also recapitulating the characteristic neuronal alterations of DS (45, 48, 49). However, unlike other models with *Scn1a* truncation mutations, *Scn1a*^A1783V^ does not affect the overall *Scn1a* mRNA or total Na_V_1.1 levels (42). Of note, missense *SCN1A* mutations are found in 34%–50% of the patients (50, 51), and the A1783V mutation was identified in multiple patients with DS (52–56).

**Figure 5:**
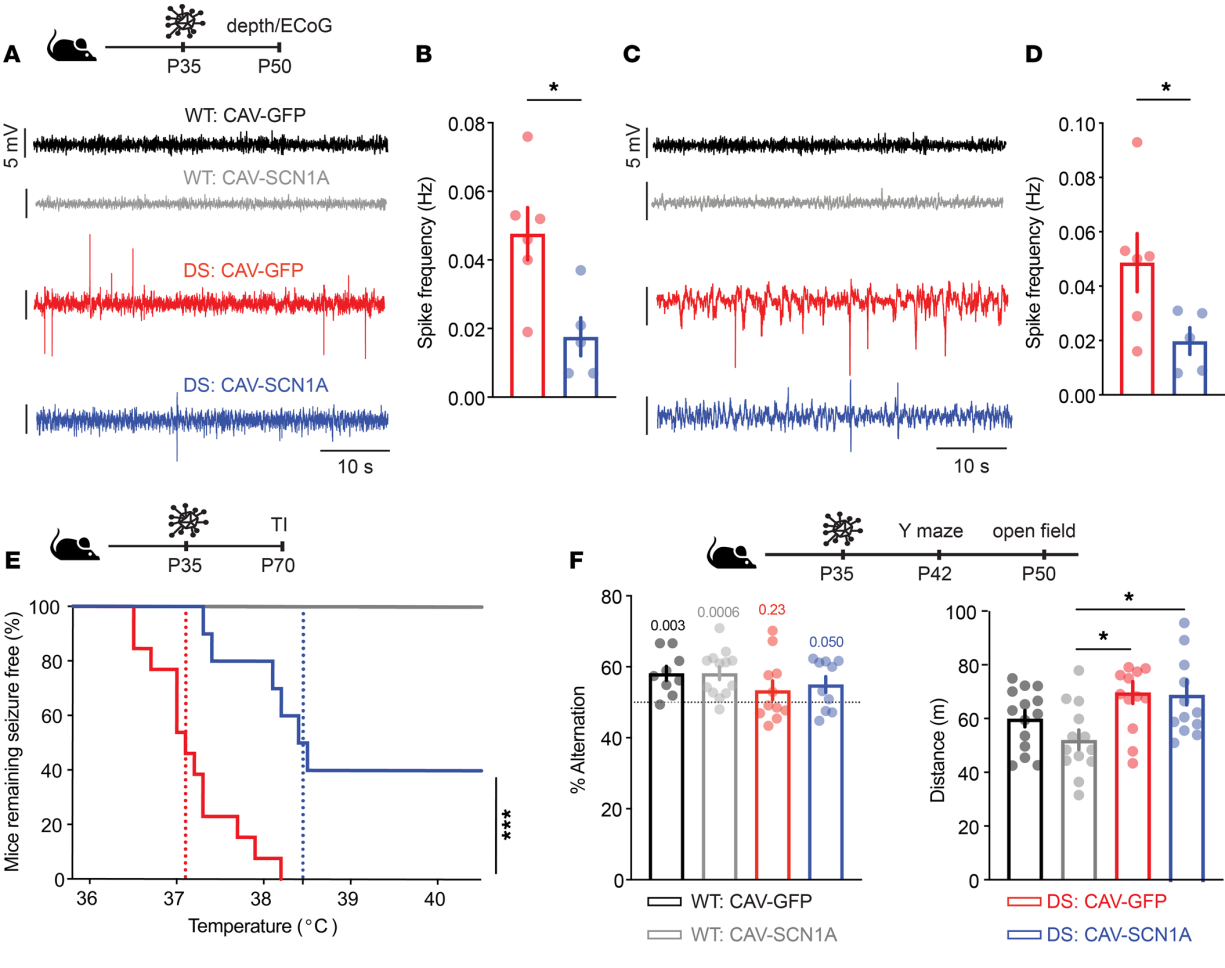
CAV-SCN1A hippocampal injections during the chronic stage reduce DS symptoms. CAV-GFP or CAV-SCN1A (1 × 10^9^ physical particles) were injected bilaterally into the hippocampi of 5-week-old WT and DS mice. Two weeks after injection, depth electrodes were implanted into the hippocampus at the site of injection. (**A**) Example traces and (**B**) quantification of the spike frequencies. (**C**) Example traces of cortical ECoG recordings. (**D**) Quantification of the spike frequencies. WT: CAV-GFP (*n* = 5) ); WT: CAV-SCN1A (*n* = 3); Epileptic activity was not detected in WT mice: CAV-SCN1A (*n* = 3); DS: CAV-GFP (*n* = 6); DS: CAV-SCN1A (*n* = 5). Statistical analyses: unpaired, 1-sample *t* test. (**E**) Mice remaining free of thermally induced (TI) seizures. The dotted lines represent the median seizure temperature. WT: CAV-GFP (*n* = 15); WT: CAV-SCN1A (*n* = 8); DS: CAV-GFP (*n* = 10); DS: CAV-SCN1A (*n* = 13). Statistical analyses: log-rank test. (**F**) Left: Spontaneous alternations in the Y maze. The dotted line signifies the chance level, expected from random alternation. The markings above the bars indicate statistical analysis using 1-sample *t* test relative to 50%. No statistical differences were observed using 2-way ANOVA. WT: CAV-GFP (*n* = 9); WT: CAV-SCN1A (*n* = 13); DS: CAV-GFP (*n* = 11); DS: CAV-SCN1A (*n* = 10). Right: The distance moved in the open field. WT: CAV-GFP (*n* = 14); WT: CAV-SCN1A (*n* = 13); DS: CAV-GFP (*n* = 13); DS: CAV-SCN1A (*n* = 13). Statistical analyses: 2-way ANOVA followed by Holm-Šidák post hoc analysis. *P < 0.05; ***P < 0.001.

Recently, vector-mediated expression of Na_V_1.1 was shown to reduce DS pathology in 5-week-old (adolescent) *Scn1a*^A1783V/WT^ DS mice (29). In that study, Mora-Jimenez et al. combined bilateral injections into 6 locations throughout the cortex, basal ganglia, and cerebellum. However, Na_V_1.1 immunoreactivity was limited to the area flanking the needle track. Considering the robust retrograde transport of CAV-2 vectors (Figures 1, 2, and 4), we asked whether injections exclusively into the hippocampi were sufficient to circumvent the epilepsy in adolescent DS mice. To that end, bilateral injections of CAV-SCN1A or CAV-GFP were performed in 5-week-old healthy (WT) and *Scn1a*^A1783V/WT^ mice. As previously reported, the prevalence of SUDEP is rare during the chronic stage (after P30) in DS mice (36, 37, 39, 42). Consistent with the above studies, only one CAV-GFP–injected DS mouse died out of the 2 cohorts (Supplemental Figure 4). In lieu of survival, we used electrocorticography (ECoG) to examine the impact of CAV-SCN1A on aberrant neuronal activity. Two weeks after injection, we implanted depth electrodes in the hippocampus and intracranial electrodes in the somatosensory cortex for ECoG recordings. Initially, these recordings showed that CAV-SCN1A injections in healthy mice did not induce aberrant neuronal activity (Figure 5, A and C). Moreover, in DS mice, CAV-SCN1A hippocampi injections reduced the occurrence of epileptic spikes (Figure 5, A–D).

Another hallmark of DS is sensitivity to thermally induced seizures. Antiseizure medications, which are effective in treating patients with DS, can elevate the threshold of thermally induced seizures in DS mice (47, 57). Strikingly, we found that approximately 40% of the CAV-SCN1A–injected DS mice showed complete protection from seizures up to 40.5°C (Figure 5E). Moreover, CAV-SCN1A injections in DS mice reduced susceptibility and elevated the temperature threshold (Figure 5E). Conversely, all the DS mice injected with CAV-GFP experienced seizure below 38.5°C (Figure 5E). Together, these data demonstrate that CAV-SCN1A injections improve the epileptic phenotypes in DS *Scn1a*^A1783V/WT^ mice during the chronic stage.

### Exogenous Na_V_1.1 activity does not alter the behavior of WT mice.

Next, we compared the behavior of CAV-GFP– and CAV-SCN1A–injected adolescent WT and DS mice in the Y maze spontaneous alternation and open field tests. The Y maze uses the natural curiosity of mice and their tendency to explore novel environments to assess working memory, while the open field test monitors activity in a novel environment. DS mice injected with CAV-GFP showed random, nondirected exploration of the Y maze as well as increased motor activity in the open field. CAV-SCN1A injections partially corrected the alternation level of DS mice, with a more directional exploration, but the mice still showed hyperactivity in the open field test (Figure 5F). Importantly, WT mice injected with CAV-GFP or CAV-SCN1A demonstrated comparable performance, with a nonrandom exploration of the Y maze and normal behavior in the open field (Figure 5F). These latter data suggest that supraphysiological levels of exogenous Na_V_1.1 activity do not have a deleterious effect on working memory in healthy mice.

### CAV-SCN1A hippocampal injections revert epileptic phenotypes in juvenile DS mice.

The severity of the epileptic phenotype subsides after the fourth week of life in *Scn1a*^A1783V/WT^ mice. Thus, we next asked whether exogenous Na_V_1.1 activity, mediated by CAV-SCN1A injections, can prevent recurrent spontaneous seizures in juvenile mice, during the severe stage (P21–P24) of the disease (39, 45). For this purpose, we performed bilateral hippocampal injections at the onset of symptoms (P21–P24). Of note, in juvenile mice, CAV-2–mediated gene transfer was robust, with mCitrine immunoreactivity throughout the hippocampus and in hippocampal projecting regions, including the neocortex and the thalamus (Supplemental Figure 7).

**Figure 6:**
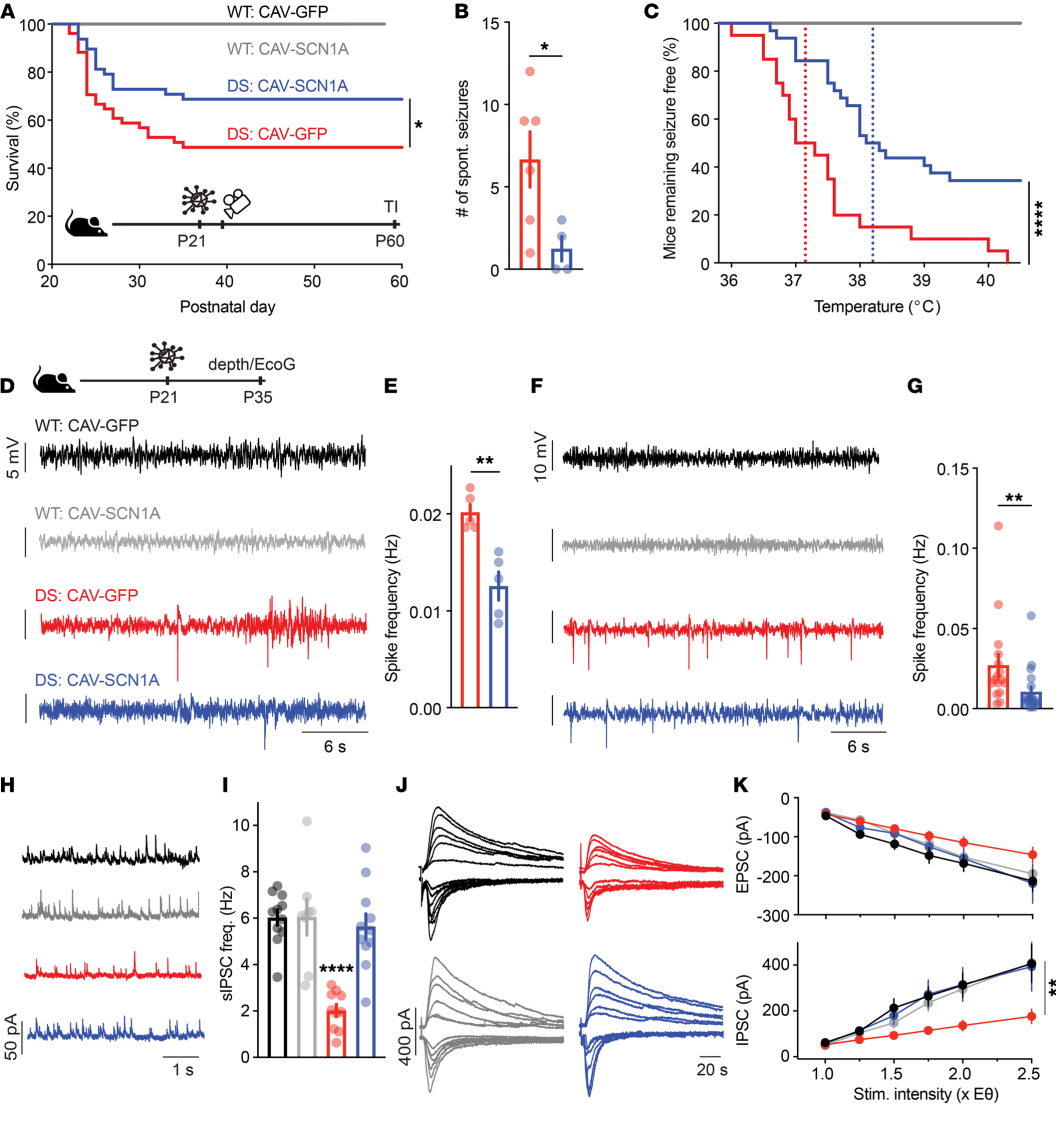
CAV-SCN1A hippocampal injection during the severe stage of DS ameliorates the epileptic phenotypes. (**A**) Survival curve of WT and DS littermates injected with either CAV-GFP or CAV-SCN1A at P21–P24 (juvenile). WT: CAV-GFP (*n* = 17); WT: CAV-SCN1A (*n* = 17); DS: CAV-GFP (*n* = 52); DS: CAV-SCN1A (*n* = 45). Statistical analyses: log-rank test. (**B**) Video monitoring of convulsive seizures 36 hours after injection. DS: CAV-GFP (*n* = 6); DS: CAV-SCN1A (*n* = 4). See Supplemental Figure 8 for additional data on individual mice. Statistical analyses: unpaired, 2-tailed *t* test. (**C**) Mice remaining free of thermally induced seizures. The dotted lines represent the median seizure temperature. DS: CAV-GFP (*n* = 20); DS: CAV-SCN1A (*n* = 32). Statistical analyses: log-rank test. (**D**–**G**) Two weeks after injection, depth electrodes (**D** and **E**) or cortical electrodes (**F** and **G**) were implanted. Example traces (**D** and **F**) and quantification of the spike frequencies are presented (**E** and **G**). DS: CAV-GFP (*n* = 5 for **E**, *n* = 16 for **G**); DS: CAV-SCN1A (*n* = 5 for **E**, *n* = 19 for **G**). No epileptic activity was detected in WT mice injected with either CAV-GFP or CAV-SCN1A (*n* = 5 for depth electrodes and *n* = 9 for cortical electrodes). Statistical analyses: Mann-Whitney test (**E**) or unpaired, 2-tailed *t* test (**G**). (**H** and **I**) example sIPSC traces (**H**) and average sIPSC frequency recorded from CA1 pyramidal neurons. Statistical analyses: 2-way ANOVA followed by Holm-Šidák post hoc analysis. (**J** and **K**) Representative traces (**J**), and average excitatory postsynaptic potentials (EPSCs) (**K**, top) and IPSCs (**K**, bottom) evoked by CA3 Schaffer collateral stimulation at different stimulation intensities. EPSCs were measured at a holding potential of –60 mV, and sIPSCs were measured at 0 mV. WT: CAV-GFP (*n* = 11); WT: CAV-SCN1A (*n* = 8); DS: CAV-GFP (*n* = 10); DS: CAV-SCN1A (*n* = 11). Statistical analyses: Mixed model repeated measures ANOVA followed by Holm-Šidák post hoc analysis. *P < 0.05; **P < 0.01; ****P < 0.0001.

In this paradigm, we found that CAV-SCN1A injections reduced (*P* = 0.034) SUDEP by approximately 40% compared with DS mice injected with CAV-GFP (Figure 6A). Short-term video recordings, 24 to 36 hours after injection of a subset of mice, demonstrated that all the mice experienced spontaneous convulsive seizures 12 hours after injection (Supplemental Figure 8). Consistent with the rapid onset of transgene expression mediated by adenovirus vectors (refs. 58, 59 and Supplemental Figure 9), by 36 hours after injection we found a reduction (*P* < 0.05) in the number of convulsive seizures in CAV-SCN1A–injected *Scn1a*^A1783V/WT^ mice, compared with DS mice injected with CAV-GFP (Figure 6B and Supplemental Figure 8).

In addition, CAV-SCN1A injections in juvenile DS mice either prevented thermally induced seizures (~30% of the mice) or increased (*P* < 0.001) the seizure threshold temperature (Figure 6C). Additionally, analyses of hippocampal and cortical recordings of a subset of DS mice 2 weeks after injection demonstrated that CAV-SCN1A injections reduced (*P* < 0.05) the number of epileptic spikes in both regions (Figure 6, D–G). Together, these data demonstrate that CAV-mediated Na_V_1.1 activity, in juvenile mice during the severe stage of DS, improved survival, reduced spontaneous seizures and epileptic spike occurrence, and increased the temperature threshold of thermally induced seizures.

### CAV-SCN1A injections restore hippocampal inhibition.

Reduced frequency of spontaneous inhibitory postsynaptic currents (sIPSCs) (11, 24, 60, 61) and reduced evoked inhibition (62, 63) likely impact DS pathology. To examine the effect of exogenous Na_V_1.1 activity on network inhibition, we used whole-cell patch clamp recordings in acute hippocampal brain slices. Consistent with the above studies, the sIPSC frequency and the amplitudes of evoked currents were low in DS mice injected with CAV-GFP (Figure 6, H–K). By contrast, 72–96 hours after injection of CAV-SCN1A, hippocampal inhibition was corrected in DS mice, with the rectification of the sIPSC frequency and evoked inhibitory current amplitudes (Figure 6, H–K). Importantly, although CAV-mediated Na_V_1.1 expression was detected in multiple types of GABAergic and excitatory neurons, enhanced evoked synaptic excitation was not observed in healthy or DS mice (Figure 6, J and K). Together, these data demonstrate that CAV-SCN1A corrects DS-associated network dysfunctions, including rapid recovery of hippocampal network inhibition. Moreover, supraphysiological levels of exogenous Na_V_1.1 activity do not deleteriously affect sIPSC frequency or evoked excitatory postsynaptic currents (EPSCs)/IPSCs in healthy mice.

### CAV-SCN1A hippocampal injections restore background ECoG activity and partially correct behavioral deficits in DS mice.

**Figure 7:**
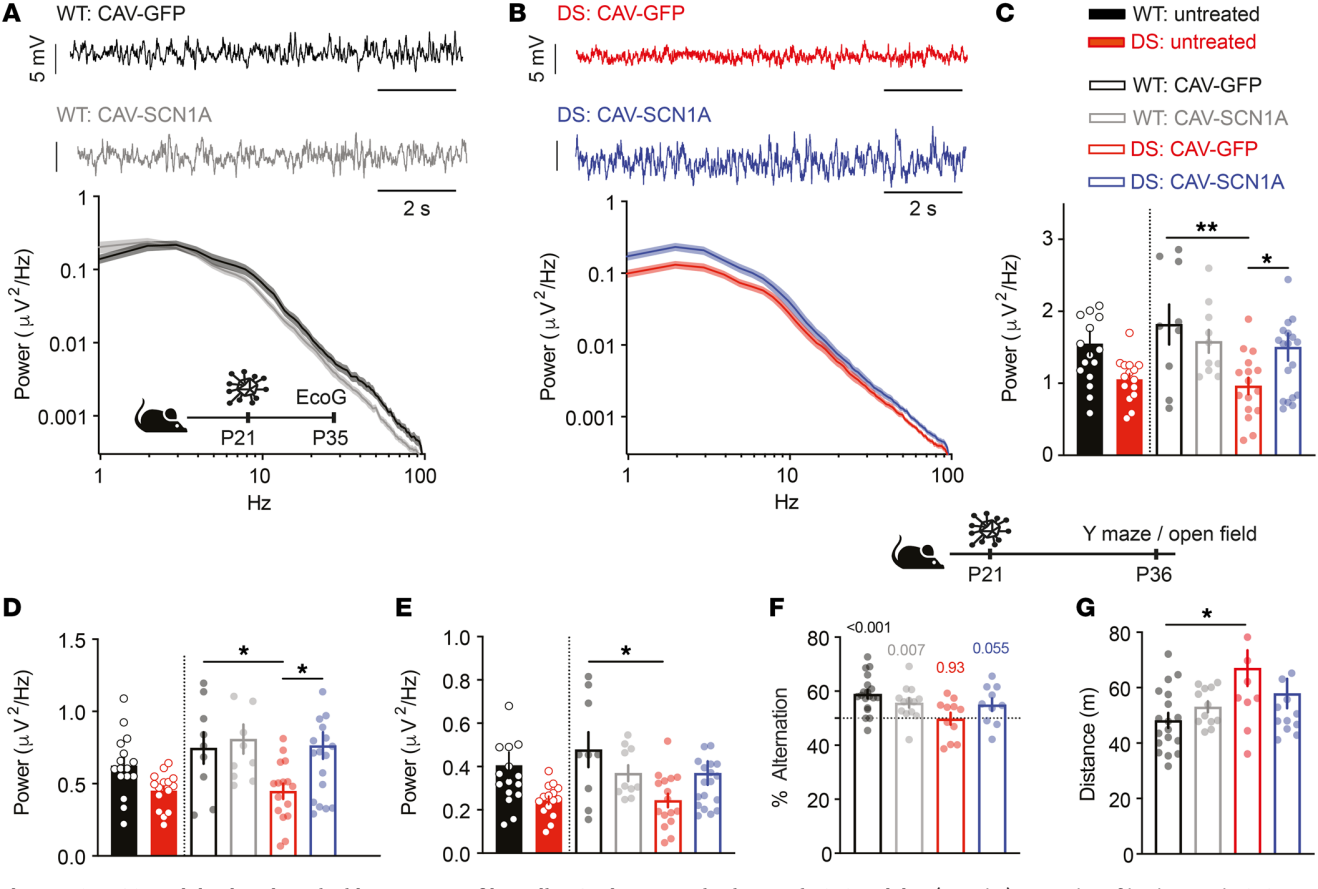
CAV-SCN1A injections into the hippocampus of juvenile DS mice correct background ECoG activity. (**A** and **B**) Examples of background ECoG traces and power density profile of WT (**A**) and DS (**B**) mice. (**C**–**E**) Total power (**C**, 0.5–100 Hz) and the power in the delta (**D**, 0.5–3.9 Hz) and theta bands (**E**, 4–8 Hz). Data for untreated mice are replotted from Fadila et al. (39) and were not included in the statistical analyses. WT: CAV-GFP (*n* = 9); WT: CAV-SCN1A (*n* = 10); DS: CAV-GFP (*n* = 16); DS: CAV-SCN1A (*n* = 19). Statistical analyses: 2-way ANOVA followed by Holm-Šidák post hoc analysis. (**F**) Spontaneous alternation in the Y maze. The dotted line signifies the chance level, expected from random alternation. The markings above the bars indicate statistical analysis using a 1-sample *t* test relative to 50%. WT: CAV-GFP (*n* = 19); WT: CAV-SCN1A (*n* = 12); DS: CAV-GFP (*n* = 11); DS: CAV-SCN1A (*n* = 10). (**G**) The distance moved in the open field. WT: CAV-GFP (*n* = 18); WT: CAV2-SCN1A (*n* = 12); DS: CAV-GFP (*n* = 11); DS: CAV-SCN1A (*n* = 13). Statistical analyses: 2-way ANOVA followed by Holm-Šidák post hoc analysis. *P < 0.05; **P < 0.01.

To further explore the impact of exogenous Na_V_1.1 activity and the potential therapeutic effect on background, nonepileptic brain oscillations, we performed spectral analysis of the ECoG signals approximately 2 weeks after injection. We found that injection of CAV-GFP or CAV-SCN1A did not alter the spectral ECoG profile of WT mice (Figure 7, A–E; data for untreated mice are replotted from ref. 39). Therefore, we concluded that neither CAV-2 vectors nor exogenous Na_V_1.1 activity, in excitatory and inhibitory neurons, impacted global brain oscillations in a healthy brain. By contrast, CAV-SCN1A injections altered the spectral ECoG profile of DS mice (Figure 7, B–E).

We have previously demonstrated that DS mice generally have lower power of background ECoG activity compared with WT mice (39). Accordingly, the power of the ECoG signals of DS mice injected with CAV-GFP was lower (*P* = 0.003) compared with that of WT mice, and similar to the power of untreated DS mice (Figure 7, C–E; data for untreated mice are replotted from ref. 39). Conversely, injection of CAV-SCN1A resulted in a higher power, particularly in the delta and theta frequency bands (Figure 7, B–E). These data indicate that exogenous Na_V_1.1 activity generated by CAV-SCN1A injections also restores ECoG activity in DS mice to WT levels.

Because of the possibility of a physiological balance between the activity of sodium channels (10, 36, 64, 65), we also assessed whether CAV-2 vector injections or exogenous Na_V_1.1 activity affected the expression of endogenous murine *Scn1a*, *Scn2a*, *Scn3a*, and *Scn8a* or the β subunits *Scn1b*, *Scn2b*, and *Scn3b*. We found that CAV-SCN1A injections did not induce long-term alterations in the mRNA level of the voltage-gated sodium channel subtypes in control or DS mice (Supplemental Figure 10).

With respect to the effect on cognitive abilities, CAV-SCN1A injections had no effect on the performance of WT mice. However, there was a tendency (*P* = 0.055) for less random exploration in CAV-SCN1A–injected DS mice (Figure 7F). Moreover, while CAV-GFP–injected DS mice traveled greater distances in the open field compared with WT mice treated with the same vector (Figure 7G), CAV-SCN1A–injected DS mice traveled a distance similar to that of WT mice (Figure 7G). We concluded that, in addition to the impact on epilepsy, CAV-SCN1A injections affected nonepileptic DS features with correction of background ECoG activity, and partial correction of the behavioral properties.

### Thalamic delivery of CAV-SCN1A reduces the epileptic phenotypes in juvenile DS mice.

**Figure 8:**
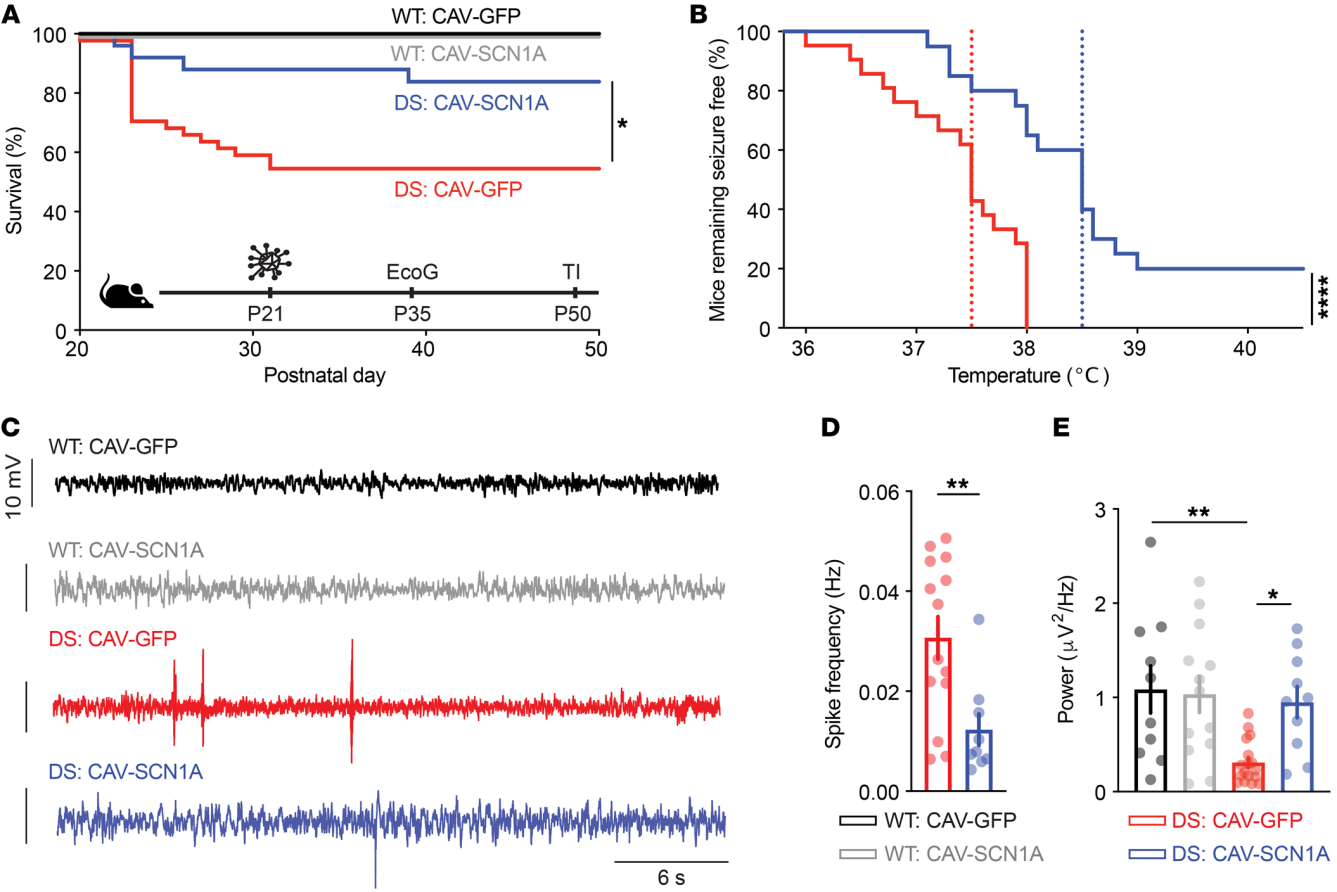
Thalamic injection of CAV-SCN1A ameliorates DS phenotypes in juvenile mice. (**A**) Survival curve of WT and DS littermates injected with either CAV-GFP or CAV-SCN1A. WT: CAV-GFP (*n* = 24); WT: CAV-SCN1A (*n* = 31); DS: CAV-GFP (*n* = 44); DS: CAV-SCN1A (*n* = 25). Statistical analyses: log-rank test. (**B**) Mice remaining free of thermally induced seizures. The dotted lines represent the median seizure temperature. DS: CAV-GFP (*n* = 21); DS: CAV-SCN1A (*n* = 20). Statistical analyses: log-rank test. (**C** and **D**) Two weeks after injection, cortical electrodes were implanted. Example traces (**C**) and quantification of the spike frequencies are depicted (**D**). Statistical analyses: Mann-Whitney test. (**E**) Total ECoG power (0.5–100 Hz). WT: CAV-GFP (*n* = 11); WT: CAV-SCN1A (*n* = 13); DS: CAV-GFP (*n* = 14); DS: CAV-SCN1A (*n* = 10). Statistical analyses: 2-way ANOVA followed by Holm-Šidák post hoc analysis. The biodistribution following thalamic injection is shown in Supplemental Figure 13. *P < 0.05; **P < 0.01; ****P < 0.0001.

In addition to the impact of the hippocampus, thalamic dysfunction contributes to network hypersynchrony and DS pathophysiology (66–70). Therefore, we asked whether thalamic injections of CAV-SCN1A could influence epilepsy in juvenile DS mice. Again, we found that CAV-SCN1A injections reduced the occurrence of SUDEP (Figure 8A) and reduced the susceptibility to thermally induced seizures (Figure 8B). Furthermore, cortical ECoG recordings demonstrated that CAV-SCN1A injections reduced the frequency of epileptic spikes (Figure 8, C and D) and corrected the power of background activity (Figure 8E). Together, these data demonstrate reduced epileptic phenotype and correction of global brain oscillations following CAV-SCN1A injection into the thalamus of DS mice.

### Thalamic and hippocampal injections further reduce epileptic phenotypes and correct spatial memory.

**Figure 9:**
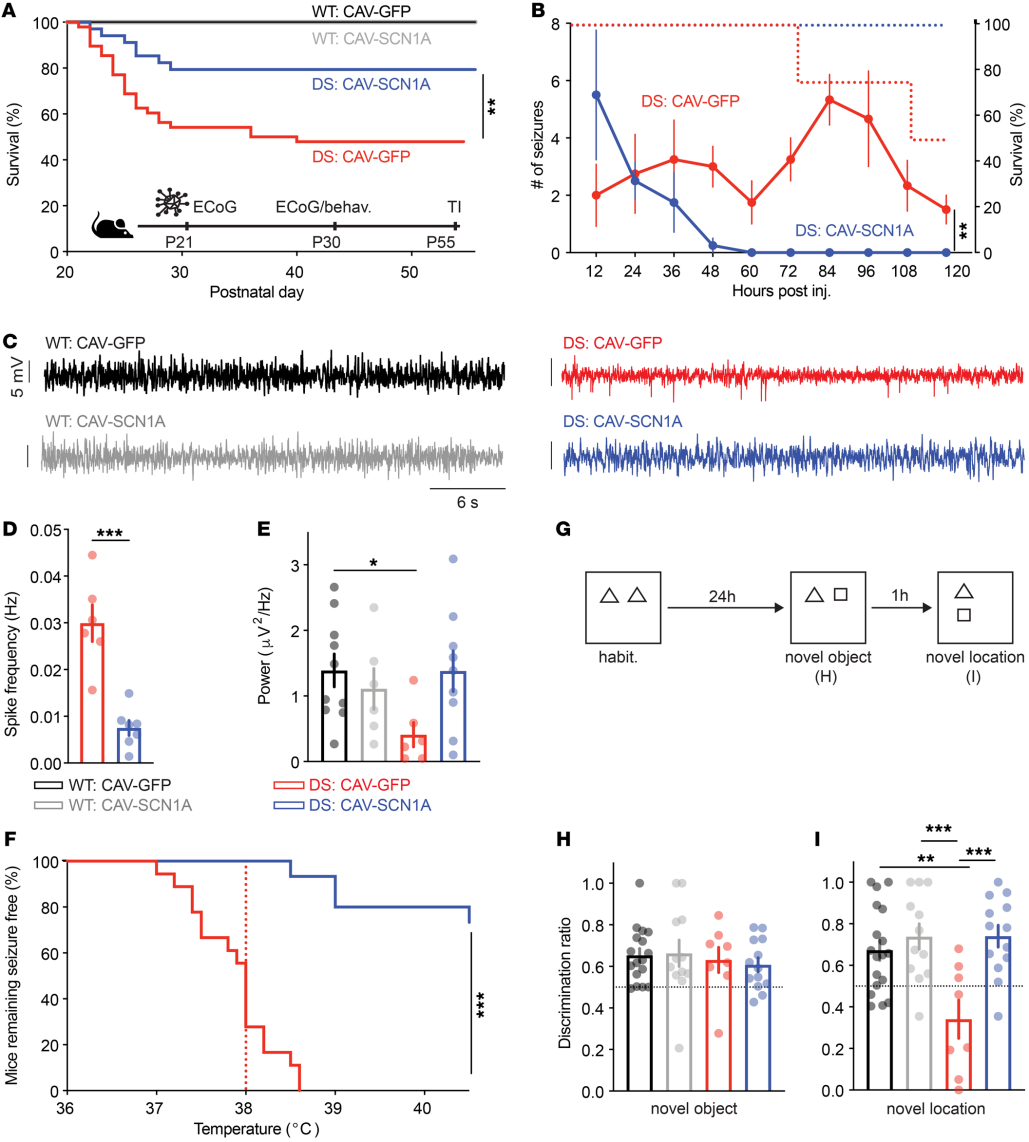
Concomitant thalamic and hippocampal injection of CAV-SCN1A in juvenile mice improves DS comorbidities. (**A**) Survival of DS mice injected with either CAV-GFP or CAV-SCN1A. DS: CAV-GFP (*n* = 48); DS: CAV-SCN1A (*n* = 31). Statistical analyses: log-rank test. (**B**) ECoG electrodes were implanted in a subset of mice, and their seizures were monitored for 5 days after injection DS: CAV-GFP (*n* = 4; 2 died, the right *y* axis depicts survival); DS: CAV-SCN1A (*n* = 4). Statistical analyses: repeated measures ANOVA. (**C**–**E**) Cortical electrodes were implanted 2 weeks after injection. Example traces (**C**) and quantification of the spike frequencies are depicted (**D**). Statistical analyses: unpaired, 2-tailed *t* test. (**E**) Total ECoG power (0.5–100 Hz) WT: CAV-GFP (*n* = 10); WT: CAV-SCN1A (*n* = 6); DS: CAV-GFP (*n* = 6); DS: CAV-SCN1A (*n* = 10). Statistical analyses: 2-way ANOVA followed by Holm-Šidák post hoc analysis. (**F**) Mice remaining free of thermally induced seizures. The dotted lines represent the median seizure temperature. DS: CAV-GFP (*n* = 18); DS: CAV-SCN1A (*n* = 15). Statistical analyses: log-rank test. (**G**–**I**) CAV-SCN1A corrects the performance of DS mice in the novel location test. (**G**) The experimental paradigm: Mice were allowed to explore the area and 2 objects for 15 minutes. Twenty-four hours later (**H**), one of the objects was changed (novel object) and mice were allowed to reexplore the area. (**I**) The third phase: 1 hour later, the novel object was moved to a new location. WT: CAV-GFP (*n* = 18); WT: CAV-SCN1A (*n* = 12); DS: CAV-GFP (*n* = 8); DS: CAV-SCN1A (*n* = 13). Statistical analyses: 2-way ANOVA followed by Holm-Šidák post hoc analysis. *P < 0.05; **P < 0.01; ***P < 0.001.

Following these results, we asked whether coinjection into the thalamus and hippocampus could further enhance the therapeutic effect of CAV-2–mediated Na_V_1.1 activity. Indeed, following dual deposits along a unique needle track, DS mice injected with CAV-SCN1A demonstrated a greater than 80% (*P* = 0.007) increase in survival (Figure 9A). To trace the frequency of electrographic seizures, ECoG electrodes were implanted in a subset of mice after vector injection and the mice were followed for 5 days. We found that all mice experienced multiple seizures 24 hours after injection. Spontaneous seizures continued in DS mice injected with CAV-GFP throughout the surveillance period. Conversely, following CAV-SCN1A injections, a reduction in seizure frequency was observed 48 hours after injection, and spontaneous seizures stopped at approximately 60 hours (Figure 9B). Moreover, similar to the effect following hippocampal or thalamic administration, coinjection of CAV-SCN1A reduced the number of epileptic spikes and tended to increase the power of background ECoG (Figure 9, C–E), and provided notable protection from thermally induced seizures (Figure 9F).

Next, we examined the ability of thalamic and hippocampal injections of CAV-SCN1A to correct cognitive functions in DS mice. Seven to 10 days after injection, mice were subjected to the novel-object location test. While DS mice injected with CAV-GFP did not demonstrate a preference to explore the object in its novel location, DS mice injected with CAV-SCN1A performed similarly to WT mice, with increased exploration of the moved object (Figure 9, G–I). Finally, the effectiveness of CAV-mediated Na_V_1.1 expression was tested in a second DS mouse harboring the R613X nonsense mutation (*Scn1a*^R613X/WT^; refs. 62, 71). Similarly to *Scn1a*^A1783V/WT^ mice, CAV-SCN1A injections improved survival and reduced susceptibility to thermally induced seizures in *Scn1a*^R613X/WT^ mice (Supplemental Figure 14).

Together, these data demonstrate that treatment of two mouse models of DS with CAV-SCN1A in the hippocampus and thalamus reduces their epileptic phenotypes and corrects DS-associated spatial memory deficits.

## Discussion

DS is an intractable childhood developmental epileptic encephalopathy, with a high fatality rate compared with other developmental epilepsies. SUDEP is the leading cause of death, with most fatalities occurring before the age of 10 years old (72). Importantly, despite polytherapy and recent advances in therapeutic options, pharmacological seizure control in DS remains notoriously difficult (73). Therefore, there is an urgent need for novel treatments.

In addition, developmental delays, cognitive impairment, and hyperactivity greatly impact the quality of life of patients and families, and therapeutic options to address these issues are also limited. Here, we demonstrate that CAV-2–mediated expression of exogenous Na_V_1.1 activity, via a codon-modified *SCN1A* ORF, can significantly improve comorbidities in juvenile and adult DS mice, ameliorate the epileptic phenotypes, correct background ECoG activity, and improve cognitive functions. Our data in DS mice suggest that this potent treatment may be applicable during the severe and chronic stages of DS.

C57BL/6J-*Scn1a*^A1783V/WT^ mice present with a particularly severe phenotype compared with other DS mice (7, 9, 71), which may render this model more resistant to treatment. Specifically, like patients with DS, C57BL/6J-*Scn1a*^A1783V/WT^ mice experience spontaneous seizures (Figure 9B, Supplemental Figure 8, and ref. 45) and thermally induced seizures that occur within the range of clinical febrile seizures (46). Furthermore, while the frequency of SUDEP is higher than the risk reported in patients (Figures 6, 8, and 9) (39, 74), this frequency does not take into account the fact that C57BL/6J-*Scn1a*^A1783V/WT^ mice do not receive antiseizure medication or emergency care for prolonged seizures. Thus, we opine that the epileptic phenotype of C57BL/6J-*Scn1a*^A1783V/WT^ mice faithfully represents the severity of DS epilepsy. Importantly, in contrast to other models that harbor *Scn1a* truncation mutations, effective gene therapy in C57BL/6J-*Scn1a*^A1783V/WT^ mice needs to overcome the endogenous levels of Na_V_1.1 and competition for the β subunits (42). Therefore, the therapeutic impact of CAV-SCN1A in C57BL/6J-*Scn1a*^A1783V/WT^ mice addresses a significant and challenging scenario — and its potential.

Notably, CAV-SCN1A was effective at multiple disease stages (Figures 5–9). This is in contrast to studies that provided gene-specific treatments during the asymptomatic, pre-epileptic stage (5, 7–9), or during the chronic phase (6, 29). Moreover, adenovirus vectors lead to transgene expression within hours after injection (58, 59), in contrast to most AAV vectors, which take between 10 and 14 days for expression (75, 76). While posttranslational modifications and trafficking obviously impact functional readouts, CAV-2–mediated transgene expression can be detected in less than 3 hours (Supplemental Figure 9), which may be advantageous for treatment during the severe stage of DS (58, 59). As our approach relies on vector-mediated *SCN1A* ORF delivery, rather than transcriptional activation of a functional (and a mutated) *SCN1A* allele, it is potentially suitable for patients with truncation or missense mutations in *SCN1A*, with demonstrated efficacy in 2 DS models (Figures 5–9 and Supplemental Figure 14).

Despite our encouraging advances, further progress may be possible. Similar to the spontaneous seizures observed when other gene therapy approaches designed to enhance *Scn1a* transcription were administered on P2 (7, 8), we also detected residual epileptic activity after therapy. Of note, antisense oligonucleotide treatment given on P14, still in the midst of the pre-epileptic stage, could not achieve full protection from death (7). Similarly, epileptic spikes and spontaneous seizures were detected following neonatal dCas9-based *Scn1a* activation (5), *Scn1a* upregulation in interneurons (8), and SUDEP (despite improved survival) was still evident following vector-mediated overexpression of *Scn1b* (9). Likewise, in most cases, abnormal epileptic activity persisted when the treatments were administered during the chronic stage of the disease (6, 29).

In our approach, administration of CAV-SCN1A to the hippocampus or the thalamus had a similar effect on DS epilepsy (Figures 6 and 8), consistent with an involvement of these areas in DS pathophysiology. Concomitant delivery into these 2 structures afforded similar protection from SUDEP (Supplemental Figure 15A), but greater protection from thermally induced seizures (Supplemental Figure 15B), and improved spatial memory (Figure 9I). Interestingly, distinct circuit-specific neuronal dysfunction has been described for each of these regions. Disinhibition was proposed as the culprit in the hippocampus (22, 36, 38, 44, 45, 77, 78). Conversely, complex neuronal changes were reported in the thalamus, with reduced activity of inhibitory and excitatory neurons (67, 68, 70), as well as hyperexcitability of inhibitory thalamic reticular nucleus neurons that lead to augmented cortico-thalamic oscillations and seizures (66). Our brain slice recordings demonstrated correction of the frequency and amplitude of spontaneous and evoked inhibition onto CA1 pyramidal neurons (Figure 6, H–K). Thus, the therapeutic effect of CAV-SCN1A in both brain regions, despite possible divergent mechanisms, further highlights the potential of this approach and, critically, our limited understanding of DS pathophysiology.

One challenge for clinical CNS-targeted gene delivery is the need to transduce enough neurons within critical brain regions to trigger a global change in network activity. Our data demonstrate a pivotal role of Na_V_1.1 expression within the hippocampus and thalamus for DS therapy. Within the hippocampus, HA-SCN1A immunoreactivity was detected in inhibitory neurons (Figure 4), consistent with a robust impact on synaptic inhibition (Figure 6) and in agreement with the contribution of these cells to DS pathophysiology (20, 21, 40). In the context of CAV-2 vectors, the *NSE* promoter leads to a higher percentage of the hippocampal interneurons versus excitatory neurons (~3:1) (Figure 3). While clearly therapeutic, mechanistically, these data do not definitively identify whether this was due to Na_V_1.1 activity in inhibitory neurons, excitatory neurons, or both. Furthermore, with CAV-2 retrograde transport, it is possible that restoration of Na_V_1.1 activity in inhibitory and excitatory neurons that are linked to a network hub (79) (Figures 1–4 and Supplemental Figures 2, 7, and 13) contributes to the therapeutic effect. Along this line, we postulate that the greater protection from thermally induced seizures and correction of spatial memory following coinjection into the thalamus and hippocampus are mediated by Nav1.1 activity in several structures (Figure 9 and Supplemental Figure 15).

Nevertheless, there are limitations to our study. The decision to move forward with the *NSE* promoter was a compromise that balanced expression in inhibitory and excitatory neurons in the hippocampus (24, 25), with a lack of knowledge on the effect on exogenous Na_V_1.1 expression in excitatory hippocampal projecting neurons. Importantly, exogenous Na_V_1.1 activity has no adverse effects in WT or DS mice (Figures 5–9). Is it possible that transducing as many excitatory neocortical neurons as possible would provide greater protection? What is the minimal number of interneurons that are needed to be transduced to generate a therapeutic effect? Do we have to transduce selected populations of inhibitory neurons (e.g., PV neurons) at the site of injection? Further studies are needed to address these important open questions. Most of our analyses were performed following CAV-SCN1A administration at the onset of spontaneous seizures (P21; Figures 6–9), and the therapeutic potential in older mice during the chronic stage of DS was evaluated only following injection into the hippocampus (P35; Figure 5). Our choice to center more efforts on the severe stage of DS was based on clearer readouts at this stage of the disease, including protection from SUDEP, reduction in the frequency of spontaneous seizures, and correction of hippocampal inhibition, phenotypes that are improving due to the natural progression of DS in mice that survive to the chronic stage of DS (45, 80–83), and the assumption that earlier treatment may have a greater potential (7). Nonetheless, CAV-SCN1A reduced the frequency of interictal spikes and protected from thermally induced seizures at the chronic stage of DS (Figure 5). Another open question is the possible sexually divergent effects related to DS therapy. At the chronic stage, CAV-SCN1A provided greater protection from thermally induced seizures in males (Supplemental Figure 5). Conversely, at the severe stage, greater protection from SUDEP was observed in female mice (Supplemental Figure 11). Although a mechanistic understanding of these differences is currently limited, sex-related differences in DS mice have been reported (9, 83), and may be important for clinical translation. Lastly, there are 15 different DS mouse models (71). Here, the beneficial effect of CAV-SCN1A was mostly examined in DS mice harboring the human A1783V missense mutation (Figures 5–9), with further validation in a second model harboring the human R613X nonsense mutation (Supplemental Figure 14). Examination in additional DS mice, including models with microdeletions in *Scn1a* (35, 36), could be informative prior to trials in humans.

Nearly global brain reactivation of *Scn1a* expression in the mouse brain provided an encouraging roadmap toward DS therapy (11). However, genetically modifying all the neurons in the human brain will never be possible — or needed — for DS therapy. Here is where CAV-2 vectors can have an additional impact on the fundamental understanding of DS inception and progression. Using transcriptional and/or translation control elements, we can target Na_V_1.1 activity to specific neuronal subpopulations, in specific regions of the brain, and at a given age, to identify their role during the inception and evolution of DS. There are open questions as to whether exogenous Na_V_1.1 activity will be needed only during the severe stage or for the life of a patient, and how many neurons need to be corrected. Addressing these questions should allow us to efficiently treat more DS comorbidities. There are several additional avenues to explore, including optimizing the dose (NB: we injected only 1 × 10^9^ physical particles/hemisphere) or the inclusion of additional expression cassettes in HD CAV-2 vectors that could affect epileptic activity. Moreover, exogenous Na_V_1.1 activity may synergize with pharmacological approaches to further improve DS therapy.

While robust and durable CAV-2–mediated expression has been demonstrated in rodents, dogs, and nonhuman primates (14, 84, 85), the shadow of the immunogenicity of some human adenovirus (HAdV) vectors is difficult to escape (86). Very few vectors derived from the more than 300 adenovirus types have been used for gene transfer to the CNS. The prototype HAdV vector derived from type 5 (HAdV-5) is preferentially taken up by astrocytes and microglial cells, the resident antigen-presenting cells in the brain parenchyma, and HAdV-5–transduced cells can be readily detected by the host immune response. CAV-2 vector efficacy is due, in part, to the lack of uptake by, and activation of, glial cells. While CAV-2 vectors have numerous characteristics that make them ideal for therapies that need large or multiple expression cassettes, the capsid (~90 nm diameter), which is slightly smaller than lentivirus vectors (110 nm) but larger than AAV particles (~20 nm), limits passive diffusion (in contrast to active dispersal via retrograde axonal transport from the injection site). Yet, the CAV-2 capsid is atypical in that it is neutrally charged (87), which will facilitate diffusion and limit the binding of antimicrobial peptides (88). To date, CAV-2 vectors have not been tested in the human brain. While preclinical data cannot predict safety in humans, they support further clinical development of CAV-2 as a platform for gene transfer to the brain. Of note, CAV-2–based gene transfer to the brain will need intraparenchymal administration, as transduction of brain cells following intravenous injections has not been documented. Stereotaxic delivery of vectors or cells for Alzheimer, Parkinson, or lysosomal storage diseases (89–95) can be performed in most state-of-the-art facilities. While more demanding in terms of infrastructure and time, stereotaxic delivery allows controlled targeting and the use of significantly lower doses (potentially as low as 5 × 10^10^ particles/patient).

In conclusion, these results provide a proof of concept for the potential of CAV-mediated delivery of an *SCN1A* ORF as a therapeutic approach for children and adolescents with DS-associated *SCN1A* missense and truncation mutations.

## Methods

### Animals and vector injections.

Juvenile mice were defined as P21–P24, corresponding to the severe stage of DS. Adolescent mice were defined as P35–P36, corresponding to the chronic stage, and adult mice were defined as older than 8 weeks of age. The surgery and injection coordinates for each age group and location are detailed in the Supplemental Methods.

Data from males and females were pooled in the main figures, with separate analyses presented in Supplemental Figures 5, 6, 11, and 12.

### Vector generation.

The CAV-2 vectors used in this work were generated using a seamless ligation cloning extract (SLiCE) strategy (96). The following plasmids were obtained from Addgene: the *NSE* promoter (plasmid 50958, James Bamburg; ref. 97); the CAG promoter (plasmid 51274, Pawel Pelczar; ref. 98); hSyn promoter (plasmid 22909, Edward Callaway; ref. 99); the *Dlx5*/*6* enhancer (plasmid 83900, Gordon Fishell; ref. 28), mCitrine, mCherry (plasmid 55634), SCN1A ORF (29), and the bovine growth hormone polyA sequence. Fragments were subcloned into the E1 region of E1/E3-deleted pCAV-2. To avoid vector genome rearrangements/deletions during amplification (~50,000 vector genomes are produced/cell), we modified the *SCN1A* ORF using codon optimization algorithms. This automated step was followed by manually screening and further modification of repeat sequences mainly in the transmembrane coding regions. The vectors were propagated and purified as previously described (31, 100).

### Immunohistochemistry, immunofluorescence, in situ hybridization, and Western blot analyses.

The assays were performed as previously described (17, 32, 101). A detailed description of the antibodies and protocols can be found in the Supplemental Methods.

### Voltage clamp recording in DK cells.

DK cells were infected with 200 physical particles/cell. The recordings were made 8–12 hours after infection, as described previously (32) and in detail in the Supplemental Methods.

### ECoG and depth electrode and recordings.

Seven to 10 days following vector injections, cortical or depth electrodes were implanted as described previously (39) and in detail in the Supplemental Methods.

### Behavioral experiments and thermally induced seizures.

Behavioral experiments were performed 5–17 days after injection, and thermal challenge was done approximately 1 month after injection as described previously (39, 45), with full details in the Supplemental Methods.

### Acute brain slice recordings.

Acute brain slices were made 72–96 hours after hippocampal injection of CAV-GFP or CAV-SCN1A, as described previously (62) and in the Supplemental Methods.

### Statistics.

Statistical analyses were performed using GraphPad Prism 9.2, utilizing log-rank test, 2-way ANOVA, unpaired 2-tailed *t* test, or the Mann-Whitney test, as appropriate. The tests and *P* values that were used for each panel are specified in Supplemental Table 1. Data are depicted as mean ± SEM. A *P* value of less than 0.05 was considered significant.

### Study approval.

All animal experiments were approved by the Ethical Committee for Animal Testing (Comité régional Languedoc-Roussillon) and the Institutional Care and Use Committee of Tel Aviv University. Animal handling was conducted in accordance with the European Council directive (2010/63/EU) and the ARRIVE guidelines.

## Author contributions

SF, BB, and IGDR designed and carried out the experiments, performed data analysis, and helped construct the manuscript. AM, MB, CI, and KA carried out some experiments and provided technical support. EMG, AR, and RHA designed some experiments and provided critical analyses. EJK and MR coordinated this study, secured funding, designed the experiments, and wrote the manuscript. All authors approved the final manuscript.

## Supplementary Material

Supplemental data

Supporting data values

## Version 1 05/16/2023

In-Press Preview

## Version 2 06/15/2023

Electronic publication
