# Supplementary material for: Viral vector–mediated expression of NaV1.1, after seizure onset, reduces epilepsy in mice with Dravet syndrome
Source: J Clin Invest. 2023 Jun 15;133(12):e159316. doi: 10.1172/JCI159316 (PMC10266792; doi:10.1172/JCI159316)
Supplement: Supplemental data [file jci-133-159316-s132.pdf]

Supplemental Materials

Viral vector-mediated expression of $Na_V1.1$, after seizure onset, reduces epilepsy in mice with Dravet syndrome

Saja Fadila, Bertrand Beucher, Iria G Dopeso-Reyes, Anat Mavashov, Marina Brusel, Karen Anderson, Caroline Ismeurt, Ethan M Goldberg, Ana Ricobaraza, Ruben Hernandez-Alcoceba, Eric J Kremer, Moran Rubinstein

Supplemental Methods 2

Supplemental Figures (1-15) 11

Supplemental Table 1. 27

Uncut Western blot gels 30

Supplemental References 31

Supplemental Methods

Study approval

All animal experiments were approved by the Ethical Committee for Animal Testing (Comité régional Languedoc-Roussillon) and the Institutional Care and use Committee of Tel Aviv University.

Animals

WT and DS mice harboring the global *Scn1a*$^{A1783V/WT}$ mutation were generated by crossing conditional floxed *Scn1a*$^{A1783V/WT}$ males (The Jackson Laboratory; stock #026133, C57BL/6J) with CMV-Cre females (The Jackson Laboratory; stock #006054, C57BL/6J), as described before (1, 2). DS mice harboring the nonsense R613X on the mixed C57BL/6J: 129S1/SvImJ background (The Jackson Laboratory; stock # 034129) were generated as described (3).

Mice were housed in a standard animal facility at a constant temperature of 22°C, on a 12 h light/dark cycle, with *ad libitium* access to food and water.

Vector injections

Adult (8-14 weeks) WT C57BL/6J mice were anesthetized with 90 mg/kg ketamine,10 mg/kg xylazine and 2 mg/kg of acepromazine by intraperitoneal injections resulting in deep anesthesia. The animal was positioned in the stereotaxic frame; the coordinates for the hippocampus were: anterior-posterior (AP) -2.1 mm; mediolateral (ML) ± 1.5 mm; dorsoventral (DV) -1.8 mm (4) , 2 µl containing 1×10^9 physical particles were injected on each side, using a 5 µl Hamilton syringe (0.2 µl/min)) and Leica Nanoinjector Stepper Motor Precision. Once completed, the microsyringe was left in place for 5 min before withdrawal to minimize vector leakage through the injection

tract. Analgesia was achieved with subcutaneous injections of meloxicam (5 mg/kg) at the end of the surgical procedure and repeated 24 h post-surgery.

WT and DS mice at the age of P21-24, or P35-P36 were randomly assigned for injections with CAV-NSE-GFP (CAV-GFP) or CAV-NSE-SCN1A (CAV-SCN1A). The mice were anesthetized using ketamine/xylazine (191/4.25 mg/kg), carprofen (5 mg/kg) was used for analgesia. The mice were placed in a stereotaxic device (Ultra Precise Stereotaxic Instruments, Stoelting, Wood Dale, IL, USA). A midline incision was made above the skull and holes were made using a 25G needle at the site of injection. For hippocampal injections, we used the following coordinates: AP -1.8 mm; ML ± 1.7 mm; DV -3 mm for P21-24 and AP -1.7 mm; ML ± 1.8 mm; DV -3 mm for P35-36. For thalamic injections at P21-P24 we use the following coordinates: AP -1.4 mm; ML ± 2.2 mm; DV -3.5 mm. One microliter containing 1×10^9 physical particles was injected on each side, using a 1 µl beveled needle Hamilton syringe (7000 series).

For thalamic-hippocampal co-injections, the needle was first lowered to: AP -1.8 mm; ML ± 1.8 mm; DV -3.5 mm, and a volume of 0.5 µl was injected. After a ~5 min, the injection needle was raised to DV 3 mm, and another volume of 0.5 µl was injected. The AP coordinates were slightly modified for P21 mice in which the distance between bregma and lambda was less than 3 mm, and reduced to AP -1.7 mm. The DV coordination was measured from the tip of the beveled injection needle. CAV-2 vectors were injected at a rate of 100 nl/min (Quintessential Stereotaxic Injector, Stoelting, Wood Dale, IL, USA). After injection, the syringe was kept in place for at least 5 min to prevent backflow before it was slowly retracted. The skin was then closed with sutures, and the mice were isolated for a period of 7 days according to TAU BSL-2 safety instructions.

Immunohistochemistry

Ten-days post-surgery, the animals were injected with an overdose of ketamine and xylazine and perfused transcardially with saline buffer (0.9%) followed by a fixative solution containing 4% paraformaldehyde (PFA) and phosphate buffer (PB) 0.1 M, pH 7.4. The brains were post-fixed in 4% PFA for 24 h and soaked in 30% sucrose prepared in PB 0.1 M at 4°C for at least 48 h. Next, the brains were frozen in Tissue Freezing Medium® optimum cutting temperature (O.C.T.) (MicromMicrotech, TFM-5) and stored at -80°C. OCT blocks were cut in serial 35-µm-thick coronal sections using a cryostat and collected in a solution containing glycerol and ethylene glycol in PB and kept at -80°C until used.

IHC on free-floating sections was performed as previously described (5). The following primary antibodies were used: chicken anti-GFP (1:1000, ABCAM, ab13970 RRID:AB_300798), a rat anti-HA (1:200 Roche, 11867431001) anti-GABA (Sigma-Aldrich, SAB4200721, RRID:AB_2891218); rabbit anti-glial fibrillary acidic protein (GFAP) (1:1000, DAKO, Z0334, RRID:AB_10013382); mouse anti-parvalbumin (PV) (1:500, ABCAM, ab277625); mouse anti-NeuN (1:1000, ABCAM, ab104224, RRID:AB_10711040); rabbit anti-HA (1:500 Cell Signaling Technology, mAb #3724, RRID:AB_1549585). Following the incubation with primary antibodies, sections were rinsed with Tris buffer pH 7.4 and 0.1% Triton X-100 (TBST) and incubated with the appropriated biotinylated or fluorescent secondary antibody/antibodies diluted in the blocking solution for 1 h for the biotinylated antibodies and 1.5 h for the fluorescent antibodies. The following secondary antibodies were used in this study: biotinylated donkey anti-chicken (1:500, Jackson ImmunoResearch Labs, Cat# 703-065-155, RRID:AB_2313596); Alexa Fluor® 488 donkey anti-chicken IgG (1:200, Jackson ImmunoResearch Labs 703-545-155, RRID:AB_2340375), Alexa Fluor® 555 donkey anti-rabbit IgG (1:200, Molecular Probes A

31572, RRID:AB_162543); Alexa Fluor® 647 donkey anti-mouse IgG (1:200, Molecular Probes A 31571, RRID:AB_162542); Alexa Fluor 488 donkey anti-rat IgG (1:200, Thermo Fisher Scientific Cat# A48270, RRID:AB_2896336) ; 4′,6-diamidino-2-phenylindole dihydrochloride (Sigma D8417). The sections processed for colorimetric immunohistochemistry were incubated with avidin-biotin-complex (Vector Laboratories PK-6100, RRID:AB_2336819) for 1 h at room temperature. Following washing with TBS, the peroxidase reaction was visualized using 0.05% 3,3′-diaminobenzidine (Sigma, D5637) and 0.03% hydrogen peroxide. Finally, sections were rinsed in TBS and mounted on SuperFrost Ultra Plus® slides (Epredia J1800AMNZ), dried at room temperature and counterstained using cresyl violet or hematoxylin, dehydrated and coverslipped with Eukitt (Sigma, 03989). The sections processed for immunofluorescence were rinsed in TBS and H_2Od, let dry overnight, covered using DAKO mounting media (S3023) and kept at 4°C. To test the specificity of the secondary antibodies, we omitted the primary antibodies in some sections while maintaining the rest of the procedures. All the control sections exhibited a lack of specific staining.

The colorimetric signals were visualized using a NIKON ECLIPSE NI-E and a color camera DS-Ri2 (4908*3264 px de 7.3 µm), a Leica Thunder microscope and a Leica K3C USB3 color camera (3072 X 2048 interlines, 2.4 µm pixel size) or Nanozoomer (Hamamatsu). Immunofluorescence signals were visualized using a Zeiss LSM980 Airyscan laser-scanning microscope. Images were adjusted for brightness and contrast using ImageJ. Picture setup was achieved with Adobe Illustrator CS6. Full resolution was maintained until the micrographs were cropped and assembled; at which time they were adjusted to a resolution of 300 dpi. The brain regions were identified using a mouse brain atlas (6).

In situ hybridization using RNAScope technology and immunofluorescence

RNA in situ hybridization using RNAScope technology was performed as previously described (7). We used parallel series of the ones used for IHC and immunofluorescence experiments. The free-floating sections were mounted onto SuperFrost Ultra Plus® slides (Epredia J1800AMNZ) .

The RNAscope™ Multiplex Fluorescent V2 Assay (Advanced Cell Diagnostics, Biotech, Cat. No. 323100) was used to detect *Gad1* (RNAscope® Probe- Mm-Gad1 Cat No. 400951 Manual Assay RNAscope) and *Gad2* mRNA (RNAscope® Probe- Mm-Gad2-C2 Cat No. 439371-C2 Manual Assay RNAscope), RNAscope 4-plex Negative CT probe (Cat No. 321831) and RNAscope 3-plex positive CT probes (Cat No. 320881) in the hippocampus.

On day one, slides were fixed in 4% PFA for 1 h at 4°C. Slides were then rinsed in PBS, followed by dehydration in increasing concentrations of ethanol (50%, 70%, 100% and 100%). Next, the sections were placed in the RNAScope oven at 60°C for 1 h, followed by the H_2O_2 blocking treatment and the antigen retrieval treatment included in the kit and washed in ethanol 100%. Finally, the sections were left to dry overnight at RT. On the next day, a hydrophobic barrier was drawn around the brain sections. Sections were processed for the protease treatment III (provided in the kit) for 30 min at 40°C in a humid chamber using an RNAScope oven, followed by incubation with the probes for 2 h at 40°C and incubation with preamplifier and amplifier probes (AMP1, 40°C for 30 min; AMP2, 40°C for 30 min; AMP3, 40°C for 15 min). Slides were incubated in fluorescently labeled probes by selecting a specific combination of colors associated with each channel Opal 570 for *Gad1* and Opal 690 for *Gad2* mRNAs, following the instruction provided by the kit.

Once the *in situ* hybridization was finished, the sections were processed for immunofluorescence. We used the same protocol that was described above in free-floating sections using chicken anti-

GFP (1:1000, ABCAM, ab13970 RRID:AB_300798), Alexa Fluor® 488 donkey anti-chicken IgG (1:200, Jackson ImmunoResearch Labs 703-545-155, RRID:AB_2340375) and 4′,6-diamidino-2-phenylindole dihydrochloride (Sigma D8417). After washing with TBS and H_2Od, the sections were left to dry overnight, coverslipped using DAKO mounting media (S3023) and kept at 4°C. Positive and negative controls for the RNAScope were performed in parallel.

Immunofluorescence signals were visualized using a Confocal Zeiss LSM980 Airyscan 8Y or Dragonfly spinning disk coupled to a Nikon inverted microscope. Images were adjusted for brightness and contrast by using Imaris (RRID:SCR_007370) and Fiji. Picture setup was achieved with Adobe Illustrator CS6. Full resolution was maintained until the micrographs were cropped and assembled; at which time they were adjusted to a resolution of 300 dpi.

Western blot

The hippocampus and neocortex were dissected 10 days post-injection. Crude membranes were prepared as previously described (8). Fifty microgram aliquots of crude membrane proteins were separated on Tris-acetate gel (6%) and transferred onto PVDF membrane. After overnight blocking in 5% nonfat dry milk in Tris-buffered saline (TBS), the membranes were incubated overnight with anti-HA antibody (1:100; mAb #3724, Cell Signaling Technology), anti-Na_V1.1 antibody (1:200, #ASC-001Alomone Labs) or alpha 1 Na^+/K^+ ATPase (1:200, # ANP-001, Alomone Labs), followed by 2 h incubation with HRP-conjugated goat anti-rabbit antibody (1:10,000, Sigma-Aldrich). The signal was visualized by chemiluminescent detection using ECL.

Voltage clamp recording in DK cells

DK cells (9) were infected with 200 physical particles/cell. The recordings were made 8-12 h post-incubation, as described previously (8). Briefly, recordings were made using a Sutter IPA amplifier (Sutter Instrument, Novato, CA, USA). The pipette solutions contained: 140 mM CsF, 10 mM

NaCl, 1 mM EGTA, 10 mM HEPES, 10 mM glucose, adjusted to pH 7.3 with CsOH. The external solution contained: 140 mM NaCl, 20 mM glucose, 10 mM HEPES, 1 mM MgCl2, 3 mM KCl, 1 mM CaCl2, adjusted to pH 7.35 with NaOH. The voltage dependence of activation was measured from a holding potential of -120 mV. Cells were depolarized for 20 ms to potentials ranging from -80 to +30 mV in 10 mV increments. The voltage dependence of inactivation was measured from a holding potential of -120 mV. Cells were depolarized for 500 ms to potentials ranging from -140 to +0 mV in 10 mV increments, followed by test pulses to -10 mV.

ECoG and depth electrode and recordings

Seven to ten days following vector injections, cortical or depth electrodes were implanted as previously described (2). Briefly, a midline incision was made above the skull, and fine silver wire electrodes (130 μm diameter bare; 180 μm diameter coated) were implanted. We used the previously formed injection holes for ECoG or depth electrodes. For hippocampal depth recordings, the wire electrodes (coated Platinum/Iridium wires,75 μm diameter bare) were lowered using the same stereotactic coordinates used for injection. A reference electrode was placed on the cerebellum; a ground electrode was placed behind the neck. The electrodes were connected to a Mill-Max connector and secured with dental cement before the skin was closed with sutures. Following the surgery, the mice were given two to five days to recover before recording.

Video-depth/EcoG recordings lasting 2-4 h were obtained during the light period from freely moving mice, connected to a T8 Headstage (Triangle BioSystems, Durham, NC, USA), using a PowerLab 8/35 acquisition hardware and the LabChart 8 software (ADInstrumnts, Sydney, Australia). The electrical signals were recorded and digitized at a sampling rate of 1 kHz with a notch filter at 50 Hz. The analysis was performed using LabChart 8 (ADInstrumnts, Sydney,

Australia). The ECoG signal was processed offline with a 0.5-100 Hz bandpass filter. Power spectral density was calculated using fast Fourier transform, with the Hann (cosine-bell) data window set to 50% overlap. We calculated the average of four to eight 30 s segments of the wakefulness, immobility and epileptic-free activity, but following a movement for each mouse, as determined by the video recording.

For the long-term video-EEG recordings depicted in Fig. 9B, the electrodes were implanted immediately following vector injection, and the mice were recorded for 5 days using a Neuralynx Digital Lynx data acquisition system (Neuralynx, Inc., Bozeman, MT, USA)

Thermally-induced seizures

Thermal induction was done about 1 month post-injection as described previously (1). Briefly, the mice were given 10 min to habituate to the thermal probe and the recording chamber. The baseline body temperature was measured, followed by an increase of 0.5°C every 2 min until 40.5°C or until a seizure was generated.

Behavioral analyses

Behavioral experiments were performed as described previously (2). Spontaneous alternation in the Y maze was assessed 5-14 days post-injection into the hippocampus and the open field test was conducted 9-17 days after treatment (hippocampal injection). Briefly, for the Y maze, the mice were placed in a symmetrical maze composed of three opaque white Plexiglas arms (each 35 cm L x 7.6 cm W x 20 cm H) and allowed free exploration for 10 min. For the open field test, mice were placed in the center of a square (50 x 50 cm) Plexiglas apparatus and their activity was recorded for 10 min. The novel object recognition test was performed 12-16 days after co-injection into the thalamus and hippocampus. On the first day, the mice were placed in a square (50×50 cm) Plexiglas apparatus for 15 min to allow for habituation. Then, two identical objects were

introduced (small Lego blocks) and the mice were allowed to explore these objects for 10 min. On the second day, one of the objects was swapped with a novel object (a different Lego block) and the mouse was allowed to explore both objects for 5 min. One hour late, the novel object was moved to a new location in the arena, and the mouse was allowed to explore both objects for 5 min. Exploration of the object was counted when the mouse's nose was within 3 cm of the object. Live tracking was achieved via a monochrome camera (Basler acA1300-60gm, Basler AG, Ahren, Germany) connected with EthoVision XT 13 software (Noldus Technology, Wageningen, Netherlands).

Acute brain slice recordings

Hippocampal injections of CAV-GFP or CAV-SCN1A were performed at P21. Acute brain slices were made 72 - 96 h later, as described before (10). The solution contained: 140 mM Cs-methanesulfonate, 5 mM CsCl, 2 mM $MgCl_2$, 2 mM 2 ATP-Tris, 0.2 mM Na_2-GTP, 10 mM HEPES and 5 mM QX-314. EPSCs were measured at a holding potential -60 mV, and IPSCs were measured at 0 mV. The minimal evoked response was defined experimentally as the stimulation intensity that produced a measurable current response in the recorded postsynaptic cell (1xEθ). As such, 1xEθ responses had a similar amplitude in both WT and DS. Next, EPSCs and IPSCs were measured in response to stronger stimuli (1.25-2.5xEθ).

Supplemental Figures (1-15)

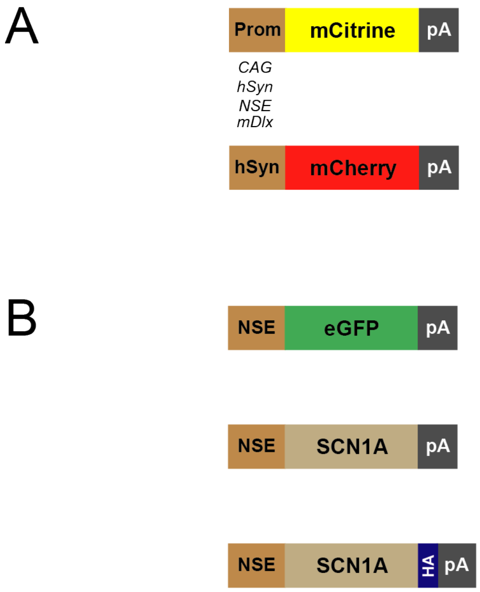

Supplemental Figure 1. **Schematics of expression cassettes used in this study**
(**A**) Prom = promoters/enhancers driving reporter cassettes: CAG, hSyn, NSE, mDlx; pA = BGHpA.
(**B**) Expression cassettes to study Nav1.1 location and therapeutic effect. CAV-GFP was used as control, the vector harboring the codon-modified *SCN1A* open reading frame (ORF) as a therapeutic vector, and the HA-tagged Nav1.1 to identify vector mediated Nav1,1 production.

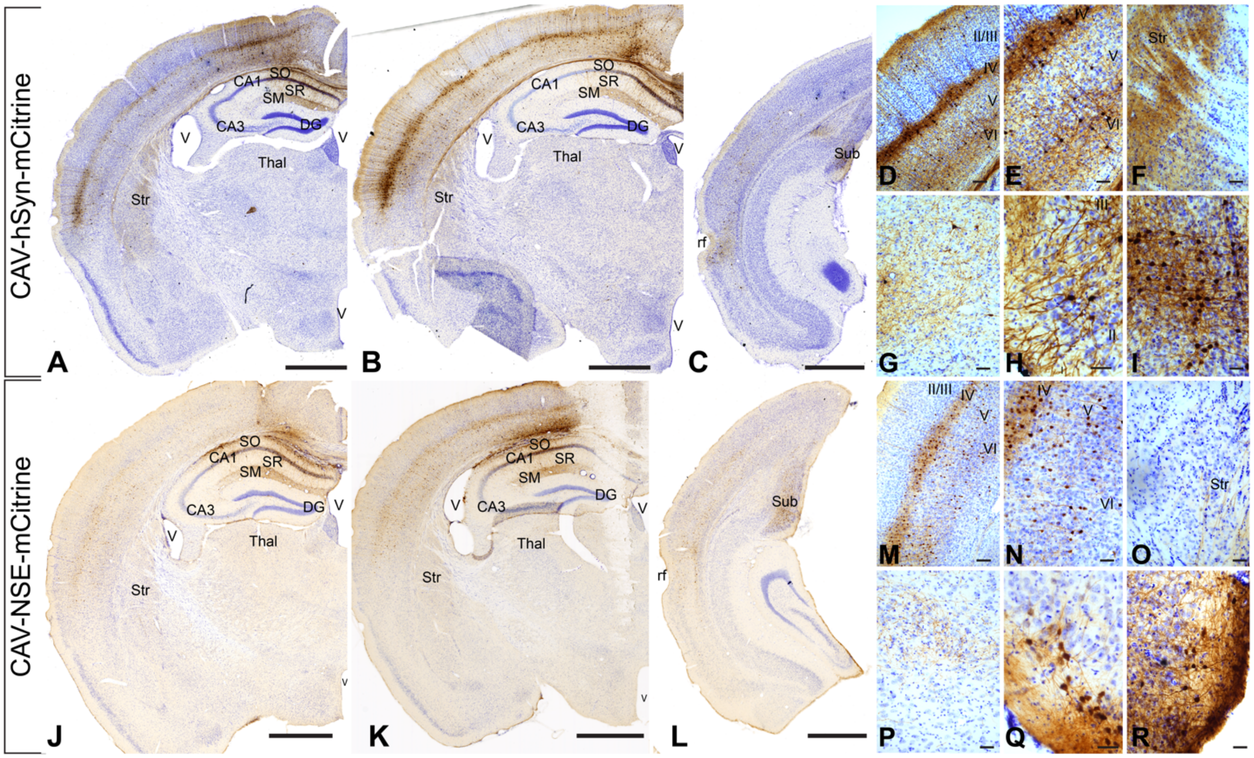

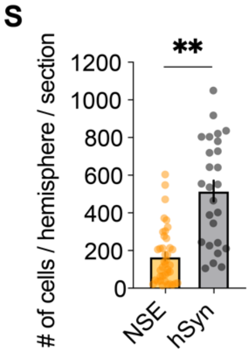

Supplemental Figure 2. Transcriptional control of transgene expression following CAV-2 vector injection into the mouse hippocampus
mCitrine immunoreactivity (brown) in several brain regions after CAV-hSyn-mCitrine (**A-I**, panel A depicts that same data shown in Fig. 1E) or CAV-NSE-mCitrine (**J-R**) injection into the hippocampus of adult mice (1 x 10^9 physical particles of each vector). All sections were counterstained using cresyl violet. (**A-C**) Low magnification showing one cerebral hemisphere. mCitrine somas and fibers were observed in the neocortex, striatum, dorsal thalamus, different hippocampal layers, entorhinal cortex and subiculum, and mCitrine immunoreactive fibers in the commisue and the external capsule. (**D-E**) High magnification of the lateral neocortex, showing the presence of mCitrine immunoreactive cells in layers II to VI.
(**F-G**) High magnifications showing the presence of mCitrine immunoreactive fibers in the striatum (**F**) and in the dorsal thalamus (**G**). (**H-I**) We also found mCitrine cells in caudal regions, corresponding to the entorhinal cortex, in layers II and II (**H**) and in the subiculum (**I**). (**J-L**) Low magnification micrographs of one cerebral hemisphere, showing the presence of mCitrine immunoreactive cells and fibers in the same regions described in A-C. (**M-N**) High magnification showing the presence of mCitrine immunoreactive cells in layer IV-VI in the lateral cortex. (**O-P**) presence of scattered fibers in the striatum (**O**) and in the dorsal thalamus (**P**). (**Q-R**) Micrographs showing mCitrine immunoreactive cells in layer II-II of the entorhinal cortex (**Q**) and in the subiculum (**R**). Scale bars: A-C: 1 mm; D: 100 µm; E-I 50 µm; J-L: 1 mm; M: 100 µm; N-R: 50 µm.
(**S**) Quantification of the transduced neocortical neurons demonstrated that CAV-NSE resulted in fewer neurons expressing the transgene. Statistical analyses used Mixed model repeated measures ANOVA to take into account that multiple sections were taken from each mouse: CAV-hSyn-mCitrine: n = 3 mice, 8 - 10 sections from each mouse; CAV-NSE-mCitrine: n = 5 mice, 8 - 9 sections from each mouse. The data in panel S (CAV-NSE) are also presented in Fig. 3E (cortex). Here the data are presented as the number of cells/hemisphere and in Fig. 3E as the number of cells/section (2 hemispheres).

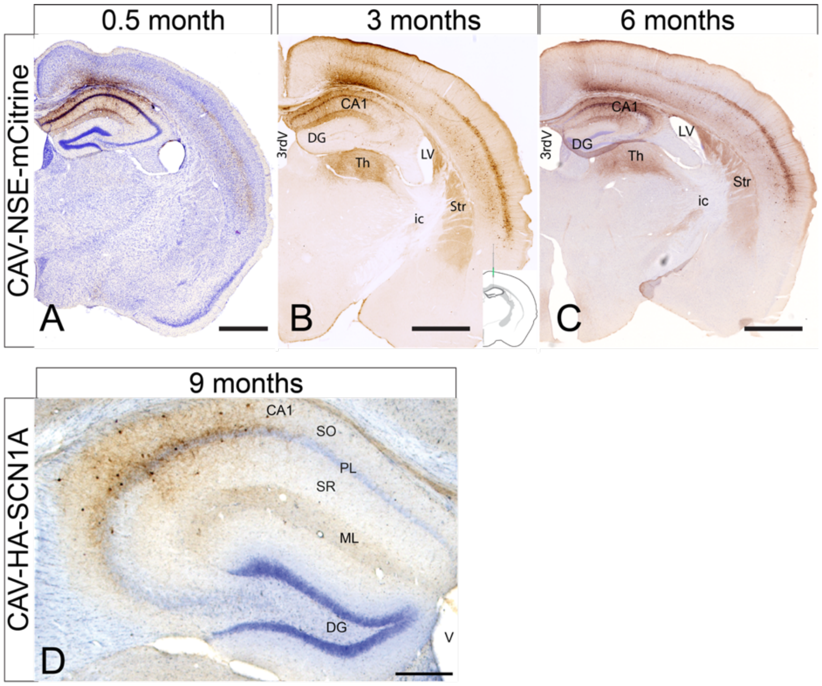

Supplemental Figure 3. Stable and robust transgene expression of CAV-2 vectors.
(**A-C**) Coronal sections of WT animals injected in the hippocampus with CAV-NSE-mCitrine, showing in brown mCitrine immunoreactivity and counterstained with cresyl violet. mCitrine immunolabeling (**A**) 2 weeks, (**B**) 3 months, and (**C**) 6 months post-injection. (**D**) CAV-HA-SCN1A was injected bilaterally into the hippocampus of adult mice. HA immunoreactivity was stable 9 months post-injection (the longest duration assayed). HA immunoreactivity is shown in brown, and counterstained using cresyl violet. Scale bar: A-C: 1 mm, D: 250 µM.

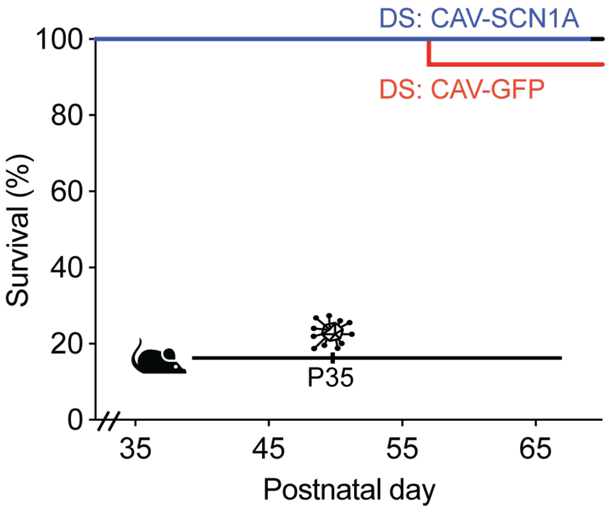

Supplemental Figure 4. Survival of adolescent mice following injection of CAV-GFP or CAV-SCN1A
Premature death in DS mice typically occurs at the 4th week of life. Here we tested the ability of CAV-SCN1A to ameliorate the epileptic phenotypes in a subset of mice that survived to the 5th week of life. As expected at this age, only one DS mouse died prematurely (treated with CAV-GFP). WT: CAV-GFP n = 14; WT: CAV-SCN1A n =13; DS: CAV-GFP n = 15; DS: CAV-SCN1A n = 13.

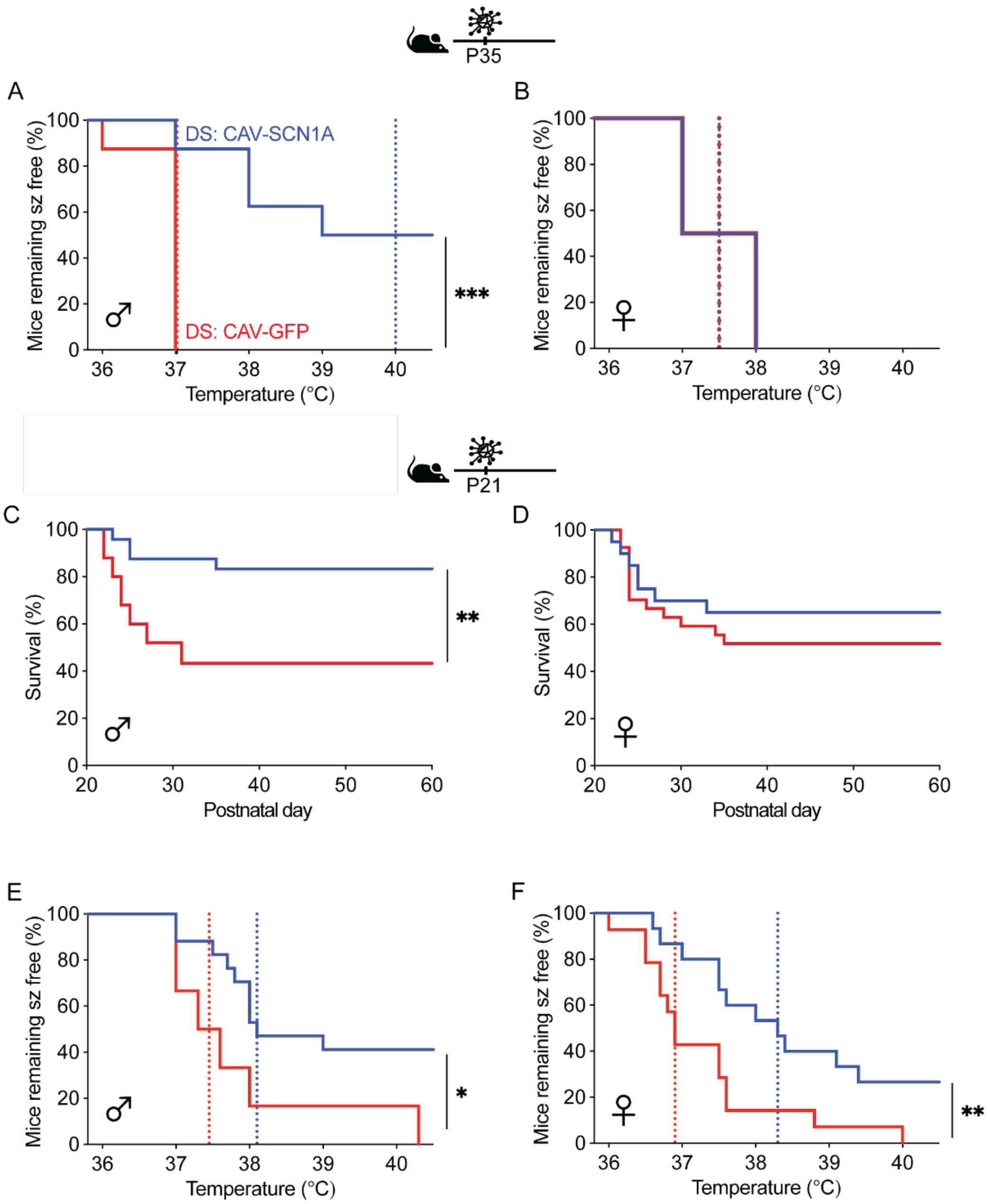

Supplemental Figure 5. **Possible sexual divergent effects following CAV-SCN1A injection into the hippocampus**
(**A-B**) Male (**A**) and female (**B**) DS mice remaining free of thermally-induced seizures following hippocampal injections of CAV-GFP or CAV-SCN1A at 5 weeks of age. The dotted lines represent the median seizure temperature. DS: CAV-GFP, ♂ n = 8, ♀ n = 2; DS: CAV-SCN1A, ♂ n = 8, ♀ n = 6.
(**C-D**) Survival curve of male (**C**) and female (**D**) DS mice injected with either CAV-GFP or CAV-SCN1A at P21. DS: CAV-GFP, ♂ n = 25, ♀ n = 27; DS: CAV-SCN1A, ♂ n = 25, ♀ n = 20.
(**E-F**) Male (**E**) and female (**F**) DS mice remaining free of thermally-induced seizures (sz). The dotted lines represent the median seizure temperature. DS: CAV-GFP, ♂ n = 6, ♀ n = 14; DS: CAV-SCN1A, ♂ n = 17, ♀ n = 15. Statistical analyses utilized the Log-rank test. *$p < 0.05$; ** $p < 0.01$; ***$p < 0.001$

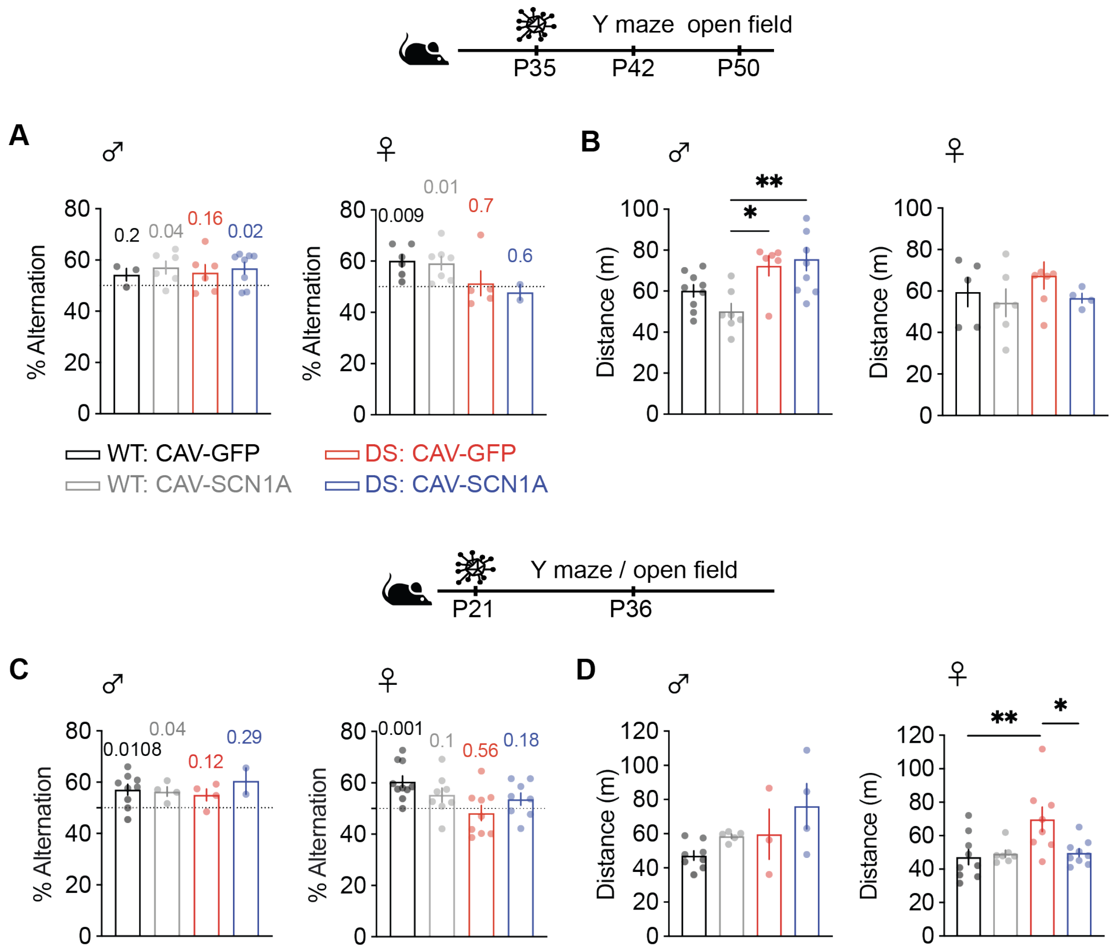

Supplemental Figure 6. Behavioral characterization of male and female mice

(**A**) Percent spontaneous alternation in the Y maze, in male and female adolescent mice (treatment at P35). The test was performed 5-10 days post-injection (median age P42, range P39-P43). The dotted line signifies chance level, expected from random alternations. The markings above the bars indicate Statistical analyses using one-sample *t* test relative to 50%. WT: CAV-GFP, ♂ n = 3; ♀ n = 6; WT: CAV-SCN1A, ♂ n = 6, ♀ n = 7; DS: CAV-GFP, ♂ n = 6, ♀ n = 5; DS: CAV-SCN1A, ♂ n = 8; ♀ n = 2.

(**B**) The open field test was performed 9-17 days post-injection (median age P50, range P45-P53). WT: CAV-GFP, ♂ n = 9, ♀ n = 5; WT: CAV-SCN1A, ♂ n = 7, ♀ n = 6; DS: CAV-GFP, ♂ n = 6, ♀ n = 7; DS: CAV-SCN1A, ♂ n = 9, ♀ n = 4. Statistical analyses utilized two-way ANOVA: ♂ main effect for genotype $p = 0.0004$ (***), main effect for vector treatment $p = 0.46$, genotype x vector treatment $p = 0.16$. The results of Holm-Sidak post hoc analysis are depicted on the graph. ♀ main effect for genotype $p = 0.45$, main effect for vector treatment $p = 0.24$, genotype x vector treatment $p = 0.67$.

(**C**) Spontaneous alternation (%) of male and female mice in the Y maze, following gene therapy in juvenile mice (P21). This test was performed 8-14 days post-injection (median age P36, range P29-P39). The dotted line signifies chance level, expected from random alternations. The markings above the bars indicate Statistical analyses using one-sample *t* test relative to 50%. WT: CAV-GFP, ♂ n = 9; ♀ n = 10; WT: CAV-SCN1A, ♂ n = 4, ♀ n = 8; DS: CAV-GFP, ♂ n = 4, ♀ n = 9; DS: CAV-SCN1A, ♂ n = 2, ♀ n = 8.

(**D**) The distance moved in the open field following gene therapy in juvenile mice. This test was performed 9-17 days post-injection (median age P37, range P30-P41). WT: CAV-GFP, ♂ n = 8; ♀ n = 9; WT: CAV-SCN1A, ♂ n = 5, ♀ n = 7; DS: CAV-GFP, ♂ n = 3, ♀ n = 8; DS: CAV-SCN1A, ♂ n = 9, ♀ n = 4. Statistical analyses utilized two-way ANOVA: ♂ main effect for genotype $p = 0.06$, main effect for vector treatment $p = 0.08$, genotype x vector treatment $p = 0.73$. ♀ main effect for genotype $p = 0.019$ (*), main effect for vector treatment $p = 0.065$, genotype x vector treatment $p = 0.026$. The results of Holm-Sidak post hoc analysis are depicted on the graph.

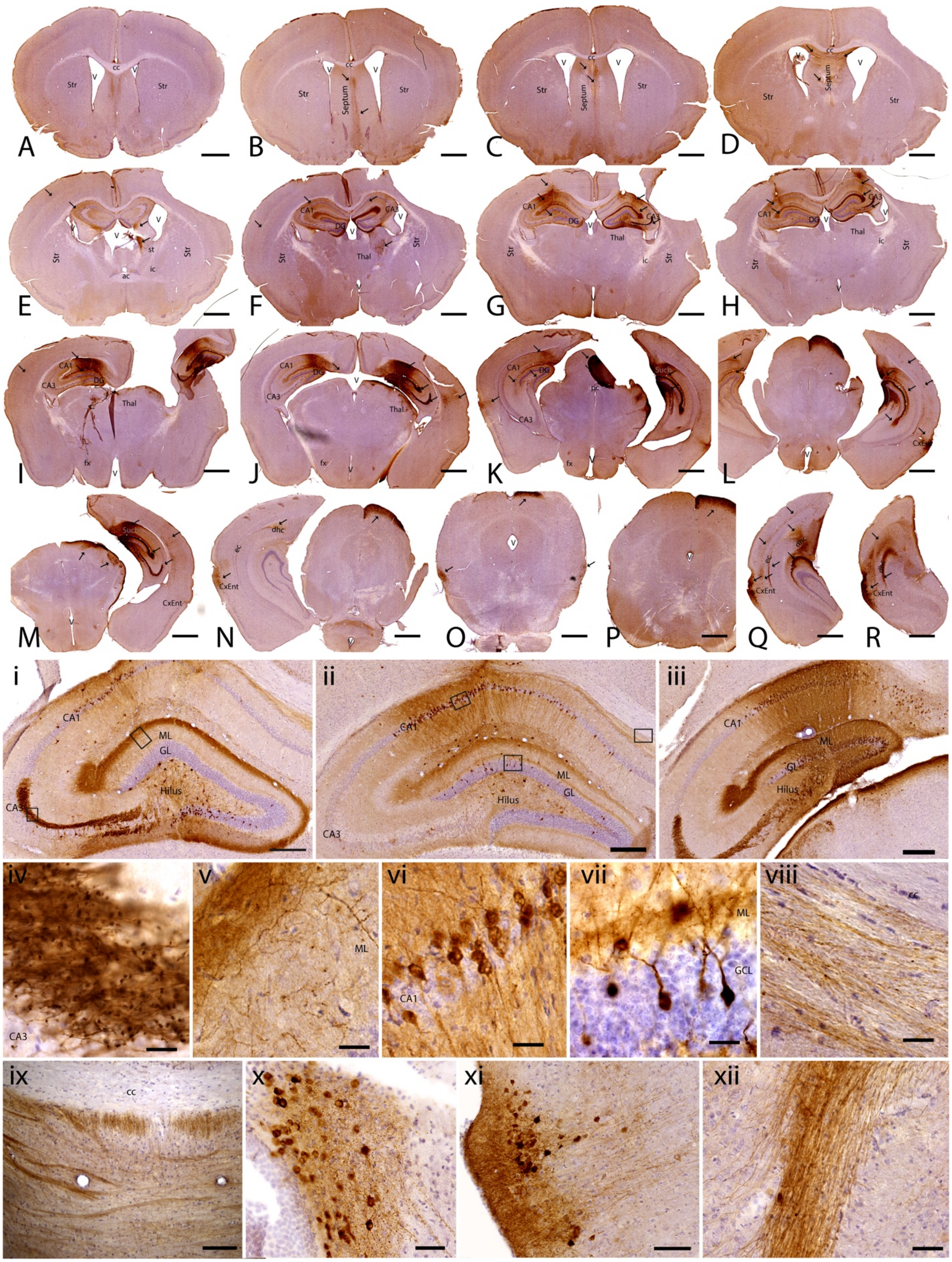
A
B
C
D
E
F
G
H
I
J
K
L
M
N
O
P
Q
R
cc
v
Str
Septum
CA1
CA3
DG
Thal
st
ac
ic
fx
pc
Such
CxEnt
dhc
ec
i
ii
iii
iv
v
vi
vii
viii
ix
x
xi
xii
ML
GL
Hilus
GCL

Supplemental Figure 7. Biodistribution of GFP in a juvenile mouse following injection of CAV-GFP into the hippocampus

(**A-R**) GFP immunoreactive cells and fibers in a representative DS mouse injected with CAV-GFP at P21 into the hippocampus. Brains were processed 1-month post-injection. Background staining is hematoxylin and GFP expression is shown by immunohistochemistry DAB staining (dark brown). The arrows indicate the expression of GFP in fibers and cells from the septum **(B-D**), thalamus **(I-J)**, hippocampal regions **(E-M** and **Q-R)**, and the neocortex **(E-M)**, including entorhinal cortex (**K-N** and **Q-R**). Scale bars: 1 mm.

(i-iii) High magnifications of the hippocampus from rostro to caudal regions, showing the presence of GFP immunoreactive cells and fibers in CA1, CA3 and DG, along the different layers, including the hilus.

(***iv-vii***) Photomicrographs showing the GFP immunoreactivity in the different areas of the hippocampus.

(iv) Detailed of the boxed area in ***(i)*** at the level of the CA3, showing fibers.

(***v***) GFP immunoreactive fibers at the level of the stratum molecular, and perforant pathway (boxed region in **i**).

(vi) magnification of the CA1 (from **ii**) showing GFP immunoreactive cells.

(vii) magnification of the boxed area in the DG of ***ii***, showing the presence of GFP immunoreactive cells also in the granular cell layer of the DG.

(viii) magnification of GFP immunoreactive fibers in the corpus callosum (cc).

(ix) GFP immunoreactive fibers in the septum.

(x) GFP immunoreactive cells in the dorsal thalamus.

(xi) GFP immunoreactive cells and fibers in the entorhinal cortex.

(xii) GFP immunoreactive fibers in the in the external capsule.

Scale bars **i-iii**: 250 μm; **iv-viii**: 10 μm; **ix**: 100 μm; **x**: 50 μm; **xi**: 100 μm; **xii**: 50 μm.

This is a representative mouse out of nine that were analyzed, all of which demonstrated wide biodistribution.

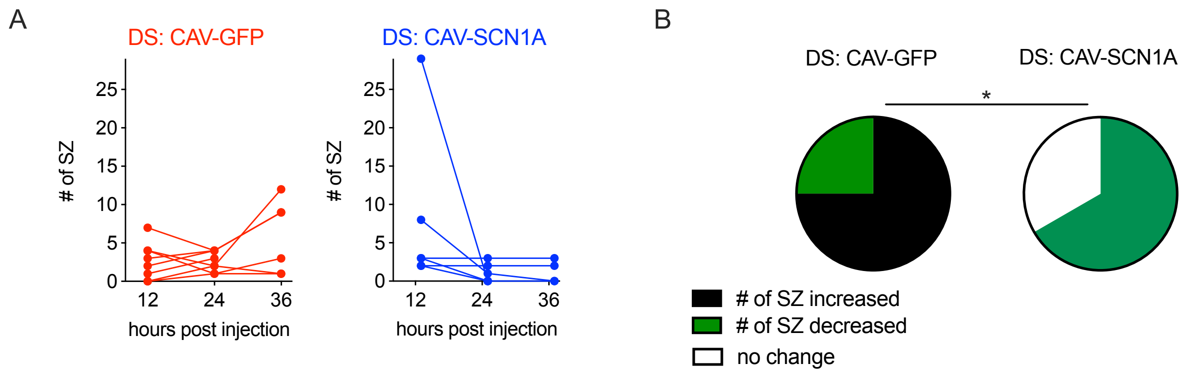

Supplemental Figure 8. Video monitoring of spontaneous convulsive seizures

A subset of mice was recorded on video for 12-36 h post-injection.

(**A**) The number of seizures (SZ) per mouse. The average number of seizures at 12 and 36 h is depicted in Fig. 6B.

(**B**) Change in the frequency of spontaneous seizures over 36 h (Chi-square test $p = 0.013$).

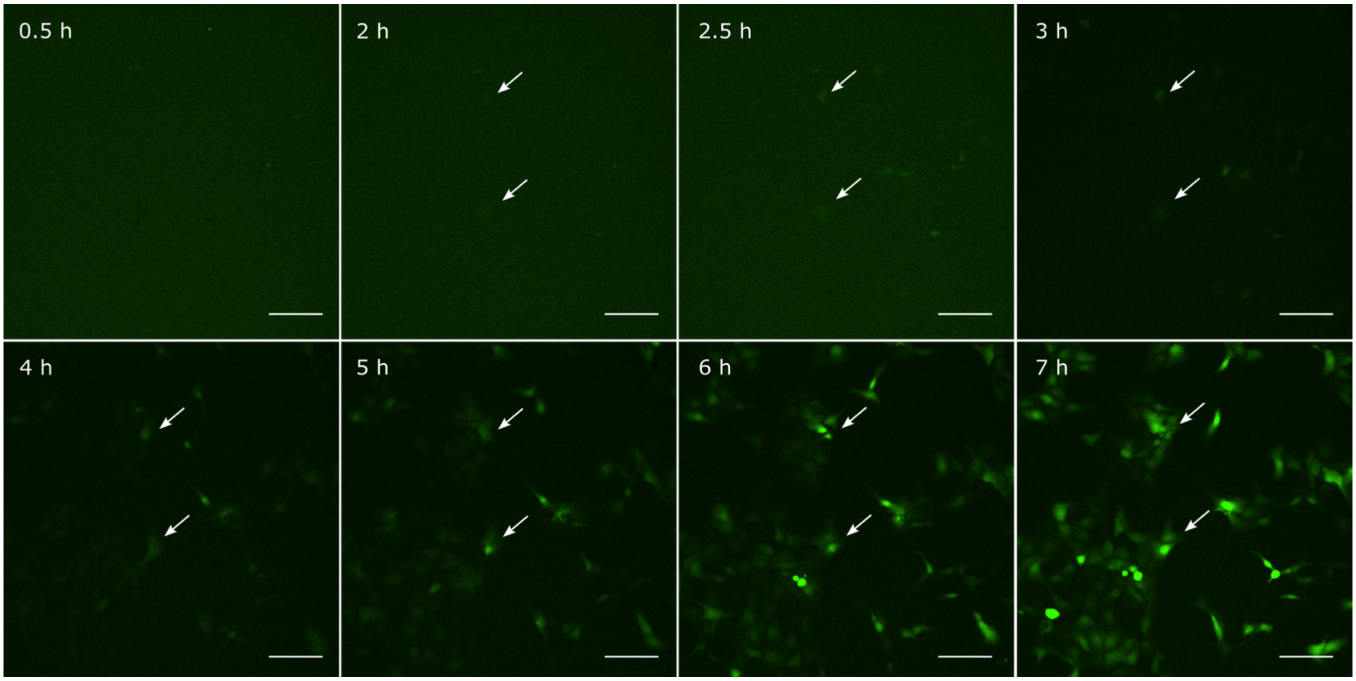

Supplemental Figure 9. Time course of mCitrine detection after incubation of DK cells with the CAV-CAG-mCitrine.

DK cells were incubated with 10 CAV-CAG-mCitrine particles/cell for ~45 min at 4°C to synchronize infection. At 4°C, CAV-2 vectors will bind to the coxsackievirus adenovirus receptor (CAR) on the cell surface, but will not be internalized. Internalization was initiated by replacing the medium with a medium at 37°C. Images of 5 fields were taken every 15 min by confocal microscopy (Nikon inverted microscope coupled to the Andor Dragonfly spinning disk) for 7 h. Detection of mCitrine beginning 2 h post-internalization (white arrows).

The contrast of the images at 0.5- 2.5 h was increased to highlight mCitrine+ cells. Scale bars: 100 µm

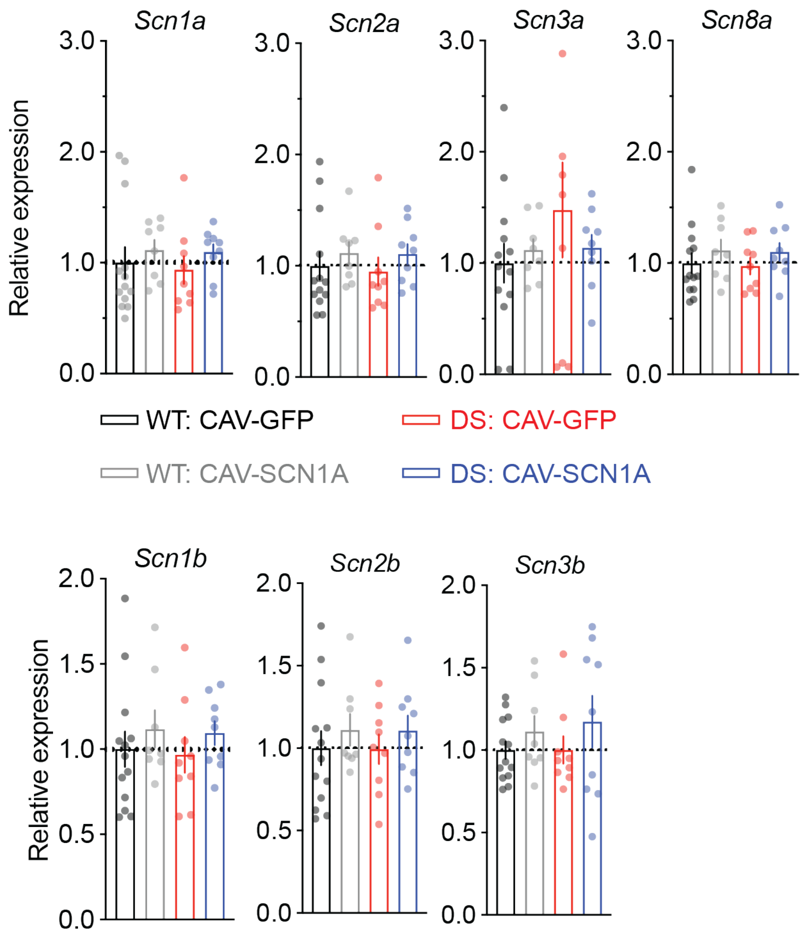

Supplemental Figure 10. Relative expression of endogenous murine voltage gated sodium channels.
CAV-GFP or CAV-SCN1A were injected into the hippocampus of P21 WT and DS mice. One-month post-injection the hippocampi were isolated and processed.
Total RNA was isolated using Purelink RNA mini kit according to the manufacturer's instructions (Thermo Fisher Scientific, Life Technologies, Carlsbald, CA, USA). cDNA was synthesized from 500 ng RNA using Maxima H Minus cDNA synthesis kit (Thermo Fisher Scientific, Life Technologies, Carlsbald, CA, USA). Real-time PCR (qPCR) reactions were performed in triplicates in a final volume of 10 µl with 5 ng of RNA as template using TaqMan gene expression assay (Applied Biosystems, Thermo Fisher Scientific, Life Technologies, Carlsbald, CA, USA) on StepOnePlus ® real-time PCR system (Applied Biosystems, Thermo Fisher Scientific, Life Technologies, Carlsbald, CA, USA). The following mRNA were quantified: *Scn1a (*Mm00450580_m1), NB our transgene was a codon-modified *SCN1A*)*, Scn2a* (Mm01270359_m1)*, Scn3a (*Mm00658167_m1)*, Scn8a (*Mm00488110_m1*), Scn1b* (Mm00441210_m1)*, Scn2b* (Mm01179204_g1)*, Scn3b (*Mm00463369_m1). Two controls were used: *Gusb (*Mm00446953_m1) and *Tfrc (*Mm00441941_m1). Efficiency of 100%, dynamic range, and lack of genomic DNA amplification were verified for all the assays. WT: CAV-GFP, n = 13; WT: CAV-SCN1A, n = 8; DS: CAV-GFP, n = 9; DS: CAV-SCN1A, n = 9.

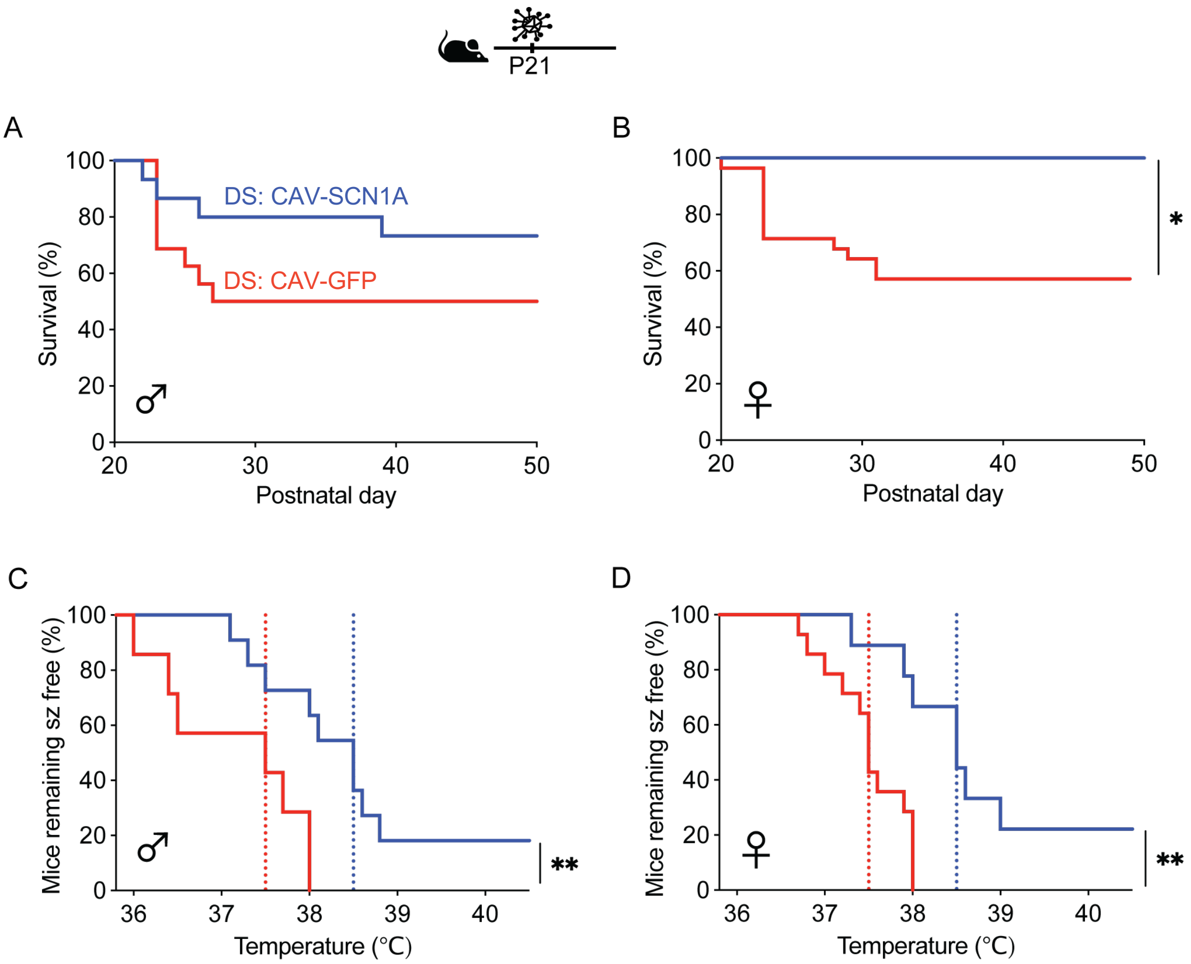

Supplemental Figure 11. **CAV-SCN1A injection into the thalamus of male and female DS mice**
(**A-B**) Survival curve of male (**A**) and female (**B**) DS mice injected with either CAV-GFP or CAV-SCN1A at P21. DS: CAV-GFP, ♂ n = 16, ♀ n = 28; DS: CAV-SCN1A, ♂ n = 15, ♀ n = 10.
(**C-D**) Male (**C**) and female (**D**) DS mice remaining free of thermally-induced seizures (sz). The dotted lines represent the median seizure temperature. DS: CAV-GFP, ♂ n = 7, ♀ n = 14; DS: CAV-SCN1A, ♂ n = 11, ♀ n = 9. Statistical analyses utilized the Log-rank test. *$p < 0.05$; ** $p < 0.01$.

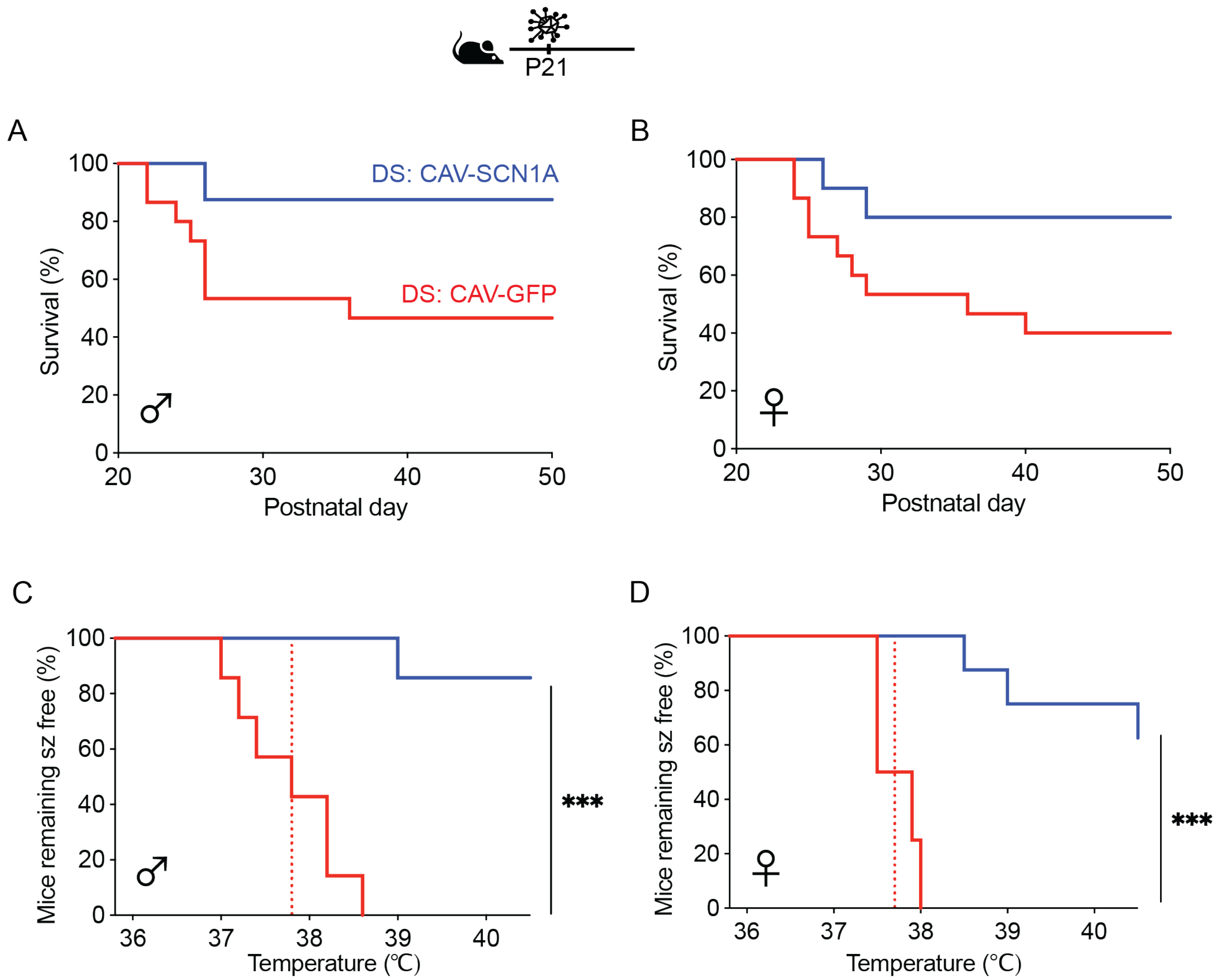

Supplemental Figure 12. CAV-SCN1A injection into the thalamus and hippocampus of male and female DS mice

(**A-B**) Survival curve of male (**A**) and female (**B**) DS mice injected with either CAV-GFP or CAV-SCN1A at P21. DS: CAV-GFP, ♂ n = 15, ♀ n = 15; DS: CAV-SCN1A, ♂ n = 8, ♀ n = 10.

(**C-D**) Male (**C**) and female (**D**) DS mice remaining free of thermally-induced seizures (sz). The dotted lines represent the median seizure temperature. DS: CAV-GFP, ♂ n = 7, ♀ n = 4; DS:CAV-SCN1A, ♂ n = 7, ♀ n = 8. Statistical analyses utilized the Log-rank test. *** $p < 0.001$.

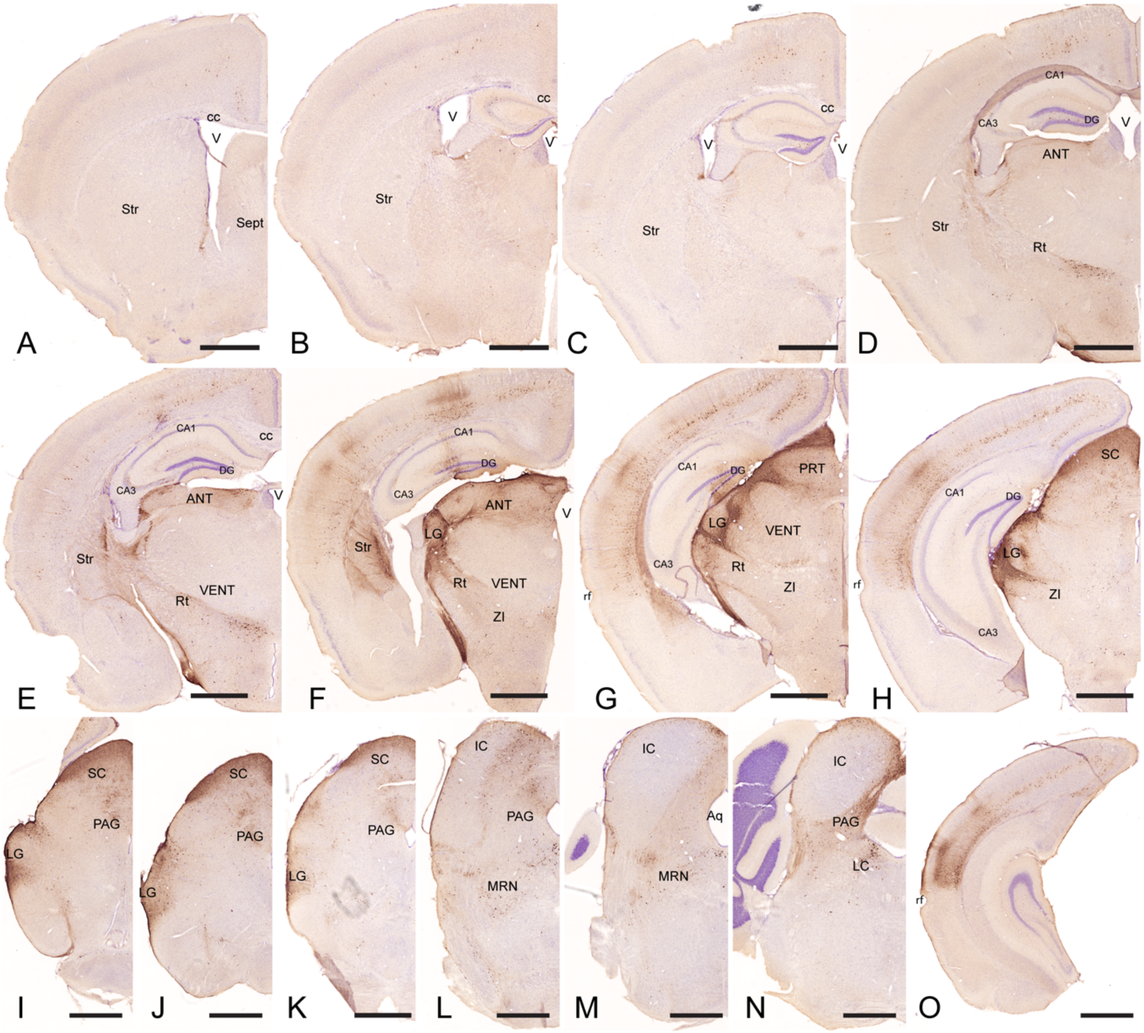

Supplemental Figure 13. Biodistribution GFP in a juvenile mouse following injection of CAV-GFP into the thalamus

GFP immunoreactive cells and fibers in a representative DS mouse injected with CAV-GFP at P21 into the thalamus. Brains were processed 1-month post-injection. GFP immunostaining is shown in brown, all sections were counterstained with Cresyl blue. Positive cells and fibers were identified in the anterior thalamus (**D-F**), ventral thalamus including the reticular nucleus and the zona incerta (**D-G**), in the lateral geniculate (**G-J**), and several regions of the midbrain and brainstem (**I-O**). In the cortex we found GFP immunoreactive cells in layer IV to VI. Scale bars: A-O: 1 mm. Str striatum; V ventricle; Sept septum, cc corpus callosum; ATN anterior thalamus; VENT ventral thalamus; Rt reticular nucleus; DG dentate gyrus; SC superior colliculus; LG lateral geniculate nuclei; PAG periaqueductal grey; IC inferior colliculus; MRN midbrain reticular nucleus; rf rhinal fissure; fi fimbria; Aq aqueduct; LC locus coeruleus; PRT pretectal

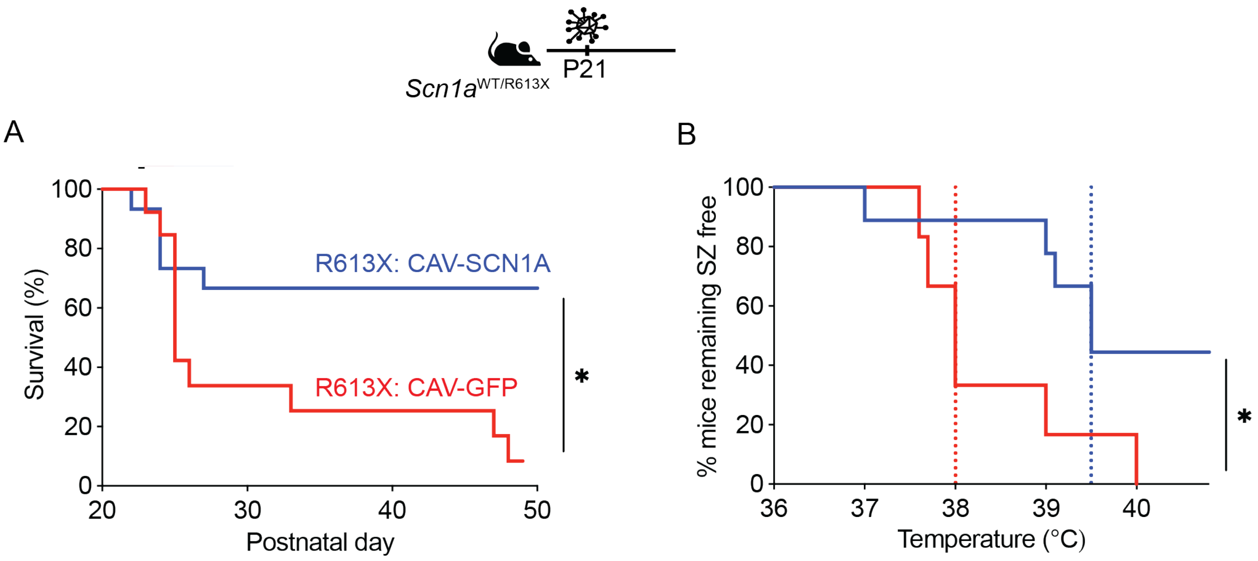

Supplemental Figure 14. **CAV-SCN1A injection into the thalamus and hippocampus is therapeutic in DS mice harboring the R613X nonsense *Scn1a* mutation**

Mice harboring the global *Scn1a*^{R613X} mutation were generated as described (3, 10). Briefly, *Scn1a*$^{R613X/WT}$ (129S1/SvImJ-Scn1aem1Dsf/J; strain 034129, The Jackson Laboratory, Bar Harbor, ME, USA), on the pure 129S1/SvImJ background were crossed with WT C57BL/6J mice (strain 000664), to generate mice on the mixed 50:50 129S1/SvImJ:C57BL/6J background. (**A**) Survival curve of DS mice. Concomitant thalamic and hippocampal injections, of either CAV-GFP or CAV-SCN1A at P21-P24 (juvenile). DS: CAV-GFP (n = 13); DS: CAV-SCN1A (n = 15). (**B**) Mice remaining free of thermally-induced seizures (SZ). The dotted lines represent the median seizure temperature. DS: CAV-GFP (n = 6); DS: CAV-SCN1A (n = 9). Statistical analyses utilized the Log-rank test. *p < 0.05

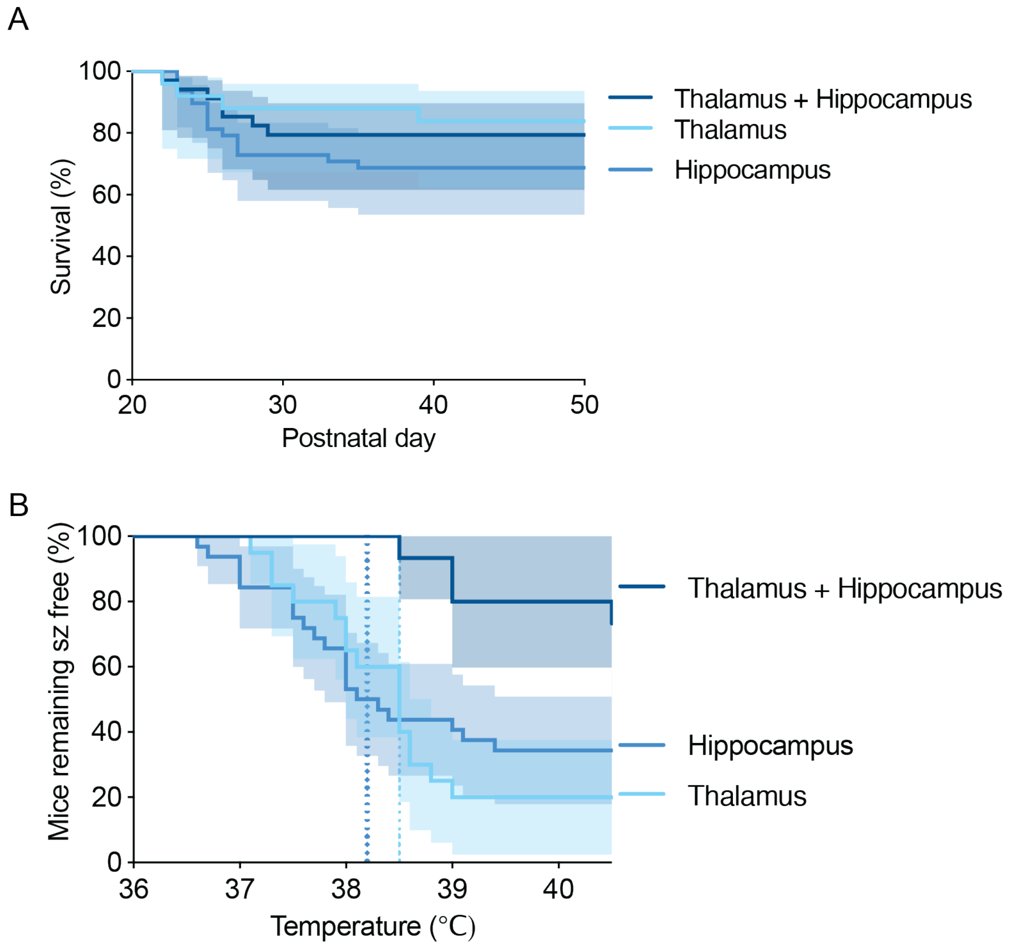

Supplemental Figure 15. **The therapeutic effect of CAV-SCN1A injections into the hippocampus, thalamus, or both, in juvenile DS mice**

(**A**) No statistical difference in the survival of DS mice following CAV-SCN1A injections into the hippocampus, the thalamus, or injections in both structures. The solid lines are the same data presented in **Figs. 6A, 8A, 9A**, respectively, and the shaded areas depict 95% confidence intervals.

(**B**) Injection of CAV-SCN1A into the thalamus and hippocampus provides greater protection from thermally-induced seizures (sz). The solid lines are the same data presented in **Figs. 6C, 8B, 9F**, respectively, the shaded areas depict 95% confidence intervals. Note the lack of overlap of 95% confidence intervals following combined injection into the thalamus and the hippocampus, demonstrating greater protection from thermally-induced seizures.

Supplemental Table 1.
Additional statistical information

Fig	Panel	Test used	*p* value and additional information
Fig. 5	B	unpaired t-test	$p = 0.013$ (*)
	D	unpaired t-test	$p = 0.048$ (*)
	E	Log-rank test	DS: CAV-GFP vs. DS: CAV-SCN1A<br>$p < 0.001$ (***)
	F<br>Y maze	One sample t-test	*p* values are marked on the graph
		Two-way ANOVA	Main effect for genotype $p = 0.079$<br>Main effect for vector treatment $p = 0.71$<br>Genotype x vector treatment $p = 0.72$
	F<br>Open field	Two-way ANOVA	Main effect for genotype $p = 0.0009$ (***)<br>Main effect for vector treatment $p = 0.32$<br>Genotype x vector treatment $p = 0.31$<br>Followed by Holm-Sidak post hoc analysis (depicted on the graph)
Fig. 6	A	Log-rank test	DS: CAV-GFP vs. DS: CAV-SCN1A<br>$p = 0.034$ (*)
	B	unpaired t-test	$p = 0.038$ (*)
	C	Log-rank test	DS: CAV-GFP vs. DS: CAV-SCN1A<br>$p < 0.0001$ (****)
	E	Mann-Whitney test	$p = 0.0035$ (**)
	F	unpaired t-test	$p = 0.0019$ (**)
	I	Two-way ANOVA	Main effect for genotype p < 0.0001 (***)<br>Main effect for vector treatment p = 0.0007 (***)<br>Genotype x vector treatment p = 0.0008<br><br>Followed by Holm-Sidak post hoc analysis (depicted)<br>GFP:WT vs. GFP:DS <0.0001 ****<br>GFP:WT vs. SCN1A:WT 0.9753 ns<br>GFP:WT vs. SCN1A:DS 0.9145 ns<br>GFP:DS vs. SCN1A:WT <0.0001 ****<br>GFP:DS vs. SCN1A:DS <0.0001 ****<br>SCN1A:WT vs. SCN1A:DS 0.9145 ns
	K	Mixed model repeated measures ANOVA	EPSCs<br>Holm-Sidak post hoc (not depicted on the graph)<br>WT-GFP vs. DS-GFP * 0.0287<br>WT-GFP vs. WT-SCN1A * 0.0181<br>WT-GFP vs. DS-SCN1A ns 0.1456<br>DS-GFP vs. WT-SCN1A ns 0.1696<br>DS-GFP vs. DS-SCN1A ns 0.1571<br>WT-SCN1A vs. DS-SCN1A ns 0.1696<br><br>IPSCs<br>Holm-Sidak post hoc (depicted)<br>WT-GFP vs. DS-GFP ** 0.0014<br>WT-GFP vs. WT-SCN1A ns 0.8018<br>WT-GFP vs. DS-SCN1A ns 0.8429

			DS-GFP vs. WT-SCN1A ** 0.0051<br>DS-GFP vs. DS-SCN1A ** 0.0017<br>WT-SCN1A vs. DS-SCN1A ns 0.8018<br><br>WT: CAV-GFP (n = 11 cells from 3 mice); WT: CAV-SCN1A (n = 8 cells from 3 mice); DS: CAV-GFP (n = 12 cells from 2 mice); DS: CAV-SCN1A (n = 11 cells from 2 mice).
Fig. 7	C	Two-way ANOVA	Main effect for genotype $p = 0.006$ (**)<br>Main effect for vector treatment $p = 0.48$<br>Genotype x vector treatment $p = 0.01$ (*)<br>Followed by Holm-Sidak post hoc analysis
	D	Two-way ANOVA	Main effect for genotype $p = 0.067$<br>Main effect for vector treatment $p = 0.87$<br>Genotype x vector treatment $p = 0.034$ (*)<br>Followed by Holm-Sidak post hoc analysis
	E	Two-way ANOVA	Main effect for genotype $p = 0.03$ (*)<br>Main effect for vector treatment $p = 0.15$<br>Genotype x vector treatment $p = 0.037$ (*)<br>Followed by Holm-Sidak post hoc analysis
	F	One sample t-test	p values are marked on the graph
		Two-way ANOVA	Main effect for genotype $p = 0.016$ (*)<br>Main effect for vector treatment $p = 0.61$<br>Genotype x vector treatment $p = 0.036$ (*)<br>Followed by Holm-Sidak post hoc analysis (depicted on the graph)
	G	Two-way ANOVA	Main effect for genotype $p = 0.007$ (**)<br>Main effect for vector treatment $p = 0.62$<br>Genotype x vector treatment $p = 0.1$<br>Followed by Holm-Sidak post hoc analysis (depicted on the graph)
Fig 8	A	Log-rank test	DS: CAV-GFP vs. DS: CAV-SCN1A<br>$p = 0.014$ (*)
	B	Log-rank test	DS: CAV-GFP vs. DS: CAV-SCN1A<br>$p < 0.0001$ (****)
	D	Mann-Whitney test	$p = 0.007$ (**)
	E	Two-way ANOVA	Main effect for genotype $p = 0.012$ (*)<br>Main effect for vector treatment $p = 0.08$<br>Genotype x vector treatment $p = 0.041$ (*)<br>Followed by Holm-Sidak post hoc analysis (depicted on the graph)
Fig. 9	A	Log-rank test	DS: CAV-GFP vs. DS: CAV-SCN1A<br>$p <0.0043$ (**)
	B	Mixed model repeated measures ANOVA	$p = 0.0037$ (**)
	D	unpaired t-test	$p = 0.0002$ (***)

	E	Two-way ANOVA	Main effect for genotype $p = 0.09$<br>Main effect for vector treatment $p = 0.218$<br>Genotype x vector treatment $p = 0.0131$ (*)<br>Followed by Holm-Sidak post hoc analysis (depicted on the graph)
	F	Log-rank test	DS: CAV-GFP vs. DS: CAV-SCN1A<br>$p < 0.0001$ (***)
	H	Two-way ANOVA	Main effect for genotype $p = 0.0977$<br>Main effect for vector treatment $p = 0.5318$<br>Genotype x vector treatment $p = 0.5385$
	I	Two-way ANOVA	Main effect for genotype $p = 0.0105$ (*)<br>Main effect for vector treatment $p = 0.0005$ (***)<br>Genotype x vector treatment $p = 0.0095$ (**)<br>Followed by Holm-Sidak post hoc analysis (depicted on the graph)

Uncut Western blot gels

Related to Figure 4S and T

Hippocmapus
a-HA antibody
(mAb #3724, Cell Signaling Technology)

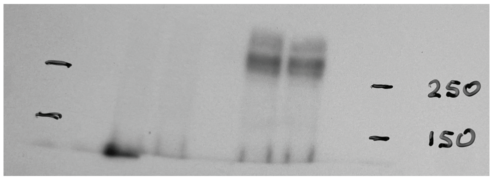

Neocortex
a-HA antibody
(mAb #3724, Cell Signaling Technology)

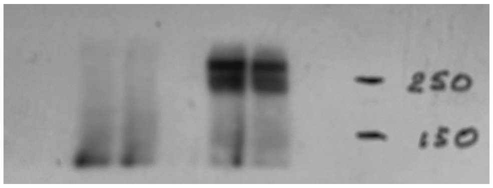

Hippocmapus
a-NaV1.1 antibody
(1:200, #ASC-001Alomone Labs)

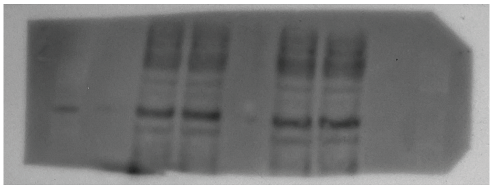

Neocortex
a-NaV1.1 antibody
(1:200, #ASC-001Alomone Labs)

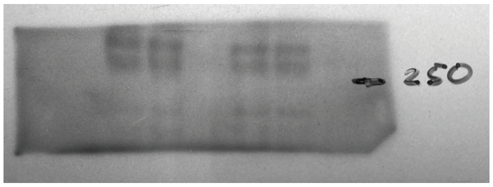

Hippocmapus
a-alpha 1 Na+/K+ ATPase antibody
(1:200, # ANP-001, Alomone Labs)

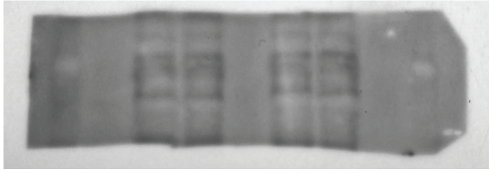

Neocortx
a-alpha 1 Na+/K+ ATPase antibody
(1:200, # ANP-001, Alomone Labs)

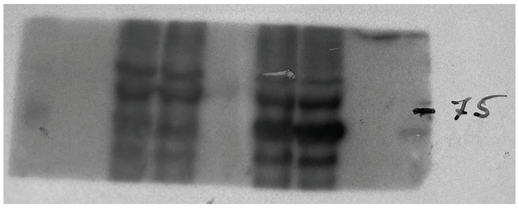

Supplemental References

1. Almog Y et al. Developmental alterations in firing properties of hippocampal CA1 inhibitory and excitatory neurons in a mouse model of Dravet syndrome. *Neurobiol. Dis.* 2021;148:105209.

2. Fadila S et al. Convulsive seizures and some behavioral comorbidities are uncoupled in the *Scn1a*^{A1783V} Dravet syndrome mouse model. *Epilepsia* 2020;61:2289–2300.

3. Mavashov A et al. Heat-induced seizures, premature mortality, and hyperactivity in a novel *Scn1a* nonsense model for Dravet syndrome. *Front. Cell. Neurosci.* 2023;17:1149391.

4. Paxinos G, Franklin KBJ. *Paxinos and Franklin's the Mouse Brain in Stereotaxic Coordinates*. San Diego: Academic Press; 2001:

5. Wehbi A, Kremer EJ, Dopeso-Reyes IG. Location of the cell adhesion molecule "Coxsackievirus and Adenovirus Receptor" in the adult mouse brain. *Front. Neuroanat.* 2020;14:28.

6. Gould TJ et al. Adaptive optics enables 3D STED microscopy in aberrating specimens. *Opt. Express* 2012;20:20998.

7. Hemonnot-Girard AL et al. Comparative analysis of transcriptome remodeling in plaque-associated and plaque-distant microglia during amyloid-β pathology progression in mice. *J. Neuroinflammation* 2022;19:1–26.

8. Nissenkorn A et al. In vivo, in vitro and in silico correlations of four de novo *SCN1A* missense mutations. *PLoS One* 2019;14:e0211901.

9. Kremer EJ et al. Canine adenovirus vectors: an alternative for adenovirus-mediated gene transfer. *J. Virol.* 2000;74:505.

10. Almog Y et al. Functional investigation of a neuronal microcircuit in the CA1 area of the hippocampus reveals synaptic dysfunction in Dravet syndrome mice. *Front. Mol. Neurosci.* 2022;15:823640.
